# The effect of various types and doses of statins on C-reactive protein levels in patients with dyslipidemia or coronary heart disease: A systematic review and network meta-analysis

**DOI:** 10.3389/fcvm.2022.936817

**Published:** 2022-07-27

**Authors:** Jie Zhang, Xinyi Wang, Wende Tian, Tongxin Wang, Jundi Jia, Runmin Lai, Tong Wang, Zihao Zhang, Luxia Song, Jianqing Ju, Hao Xu

**Affiliations:** ^1^National Clinical Research Center for Chinese Medicine Cardiology, Xiyuan Hospital, China Academy of Chinese Medical Sciences, Beijing, China; ^2^Graduate School, Beijing University of Chinese Medicine, Beijing, China; ^3^Graduate School, China Academy of Chinese Medical Sciences, Beijing, China

**Keywords:** statin, C-reactive protein, coronary heart disease, dyslipidemia, network meta-analysis

## Abstract

**Objective:**

The objective of this study was to measure the efficacy of various types and dosages of statins on C-reactive protein (CRP) levels in patients with dyslipidemia or coronary heart disease.

**Methods:**

Randomized controlled trials were searched from PubMed, Embase, Cochrane Library, OpenGray, and ClinicalTrials.gov. We followed the Preferred Reporting Items for Systematic Reviews and Meta-Analyses guidelines for data extraction and synthesis. The pairwise meta-analysis compared statins and controls using a random-effects model, and a network meta-analysis compared the types and dosages of statins using the Bayesian random-effects model. The PROSPERO registration number is CRD42021242067.

**Results:**

The study included 37 randomized controlled trials with 17,410 participants and 20 interventions. According to the pairwise meta-analysis, statins significantly decreased CRP levels compared to controls (weighted mean difference [WMD] = −0.97, 95% confidence interval [CI] [−1.31, −0.64], *P* < 0.0001). In the network meta-analysis, simvastatin 40 mg/day appeared to be the best strategy for lowering CRP (Rank *P* = 0.18, WMD = −4.07, 95% CI = [−6.52, −1.77]). The same was true for the high-sensitivity CRP, non-acute coronary syndrome (ACS), <12 months duration, and clear measurement subgroups. In the CRP subgroup (rank *P* = 0.79, WMD = −1.23, 95% CI = [−2.48, −0.08]) and ≥12-month duration subgroup (Rank *P* = 0.40, WMD = −2.13, 95% CI = [−4.24, −0.13]), atorvastatin 80 mg/day was most likely to be the best. There were no significant differences in the dyslipidemia and ACS subgroups (*P* > 0.05). Node-splitting analysis showed no significant inconsistency (*P* > 0.05), except for the coronary heart disease subgroup.

**Conclusion:**

Statins reduced serum CRP levels in patients with dyslipidemia or coronary heart disease. Simvastatin 40 mg/day might be the most effective therapy, and atorvastatin 80 mg/day showed the best long-term effect. This study provides a reference for choosing statin therapy based on LDL-C and CRP levels.

## The chemical compounds studied in this article

Atorvastatin (PubChem CID: 60823); Pravastatin (PubChem CID: 54687); Pitavastatin (PubChem CID: 5282452); Rosuvastatin (PubChem CID: 446157); Simvastatin (PubChem CID: 54454).

## Introduction

Dyslipidemia is the primary risk factor and a prerequisite for atherosclerotic cardiovascular disease (ASCVD) (1). Long-term prospective epidemiological studies have consistently demonstrated the critical role of managing dyslipidemia in reducing ASCVD morbidity and mortality (2). Nevertheless, cardiovascular events continue to occur even with a substantial reduction in low-density lipoprotein cholesterol (LDL-C) (3). Coronary heart disease (CHD) is a chronic inflammatory disease in which inflammation involves the entire process from plaque formation to rupture (4, 5). Recently, clinical trials using anti-inflammatory drugs [e.g., canakinumab (6) and colchicine (7, 8)] confirmed the direct vasculo-protective effects of primarily targeting inflammation, which may partly explain the residual risk after the normalization of LDL-C. These findings suggest that anti-inflammatory therapy provides insights into treating CHD in addition to lipid-lowering.

Statins (i.e., 3-hydroxy-methylglutaryl coenzyme A [HMG-CoA] reductase inhibitors) are used to lower cholesterol in the primary and secondary prevention of ASCVD (9). Their primary effect is to lower serum cholesterol levels by competitively inhibiting HMG-CoA reductase, thereby inhibiting hepatic cholesterol biosynthesis (9, 10). Furthermore, statins exert cardiovascular protective effects independent of lowering LDL-C (called “pleiotropic” effects) with anti-inflammatory effects that are attracting attention (11).

C-reactive protein (CRP) is a pentameric protein consisting of five identical non-covalently bound subunits of 206 amino acid residues (12). It is a major acute-phase protein in humans, a multifunctional component of the human innate host defense mechanism (12), and an indicator and predictor of ASCVD risk associated with inflammation (13, 14). CRP and high-sensitivity CRP (hs-CRP) were used for measuring the same substance, while hs-CRP is more sensitive than CRP at low CRP levels (15). The measurements include immunoturbidimetry, nephelometry, enzyme-linked immunosorbent assay, chemiluminescent enzyme immunometric assay, and radial immunodiffusion assay; a meta-analysis showed that these multiple methods could not influence the CRP results (16).

The initial statin prescription is generally based on the lipid-lowering intensity (9). It might be more beneficial if clinicians considered statins' anti-inflammatory and lipid-lowering effects (11). Previous clinical and experimental studies have shown that statins effectively reduced CRP levels (14, 17); however, comparisons of various types and doses of statins for CRP-lowering effects are inconsistent. A docking experiment *in silico* showed that rosuvastatin, fluvastatin, pitavastatin, and atorvastatin had the most substantial interactions with CRP (18). Some clinical trials demonstrated that various types and dosages of statins showed differing effects on lowering CRP levels (19, 20). In contrast, other trials found no significant differences among several statin therapies (21–23). Therefore, this study aimed to assess the effect of different types and dosages of statins on lowering CRP levels using a pairwise and network meta-analysis (NMA).

## Methods

We followed the Preferred Reporting Items for Systematic Reviews and Meta-Analyses (PRISMA) guidelines (24) for this study, and the PRISMA checklist is listed in Supplementary Table 1. This study was registered on PROSPERO (CRD42021242067).

### Eligibility criteria

We included studies that met the following criteria: (1) randomized controlled trials (RCTs); (2) participants with dyslipidemia and/or CHD (including stable angina pectoris and acute coronary syndromes); (3) studies comparing patients treated with statin vs. placebo, blank, or other types or doses of statins; (4) studies providing sufficient information on the baseline and follow-up CRP or hs-CRP level; (5) participants who were taking statins with a fixed-dose once a day; and (6) studies published in English.

The exclusion criteria were as follows: (1) Participants suffering from autoimmune diseases, malignant tumors, liver failure, kidney failure, acute inflammation, or during a perioperative period; (2) participants who were taking statins before enrollment and did not experience a washout period; (3) intervention duration of <8 weeks; and (4) fewer than 30 people per arm in the study.

### Search strategy

We searched PubMed, Embase, Cochrane Library databases, OpenGray, and ClinicalTrials.gov for eligible studies from the inception to April 1, 2021. We used a combination strategy of keywords and MeSH keywords, including “dyslipidemia,” “hypertriglyceridemia,” “hypercholesterolemia,” “coronary heart disease,” “atherosclerosis,” “atherosclerotic,” “hydroxymethylglutaryl-CoA reductase inhibitors,” “atorvastatin,” “fluvastatin,” “lovastatin,” “pravastatin,” “rosuvastatin,” “simvastatin,” “pitavastatin,” “C reactive protein,” “CRP,” and “hs-CRP.” We also scanned the references of included studies and published systematic reviews to avoid omissions. Supplementary Text 1 displays the detailed search strategy.

### Literature screening and data extraction

Two authors (WT and TXW) independently screened studies and extracted data. Disagreements were resolved through discussions with a third investigator (HX). We recorded the publication information (first author's name and year), characteristics of trials (design, location, and registration), participants (age, sex, sample size, and disease), interventions (types, dosage, and duration), other treatments, and outcomes (CRP/hs-CRP and its measurement). If possible, we extracted the results from the intention-to-treat analysis.

### Risk of bias assessment and quality assessment

Two authors (WT and TXW) independently evaluated the risk of bias according to the Cochrane Collaboration Recommendations assessment tools (25). Because CRP is an objective indicator uninfluenced by allocation concealment and blinding, it is rated as low risk regardless of allocation concealment and blinding (25). Thus, we assessed the risk of bias from the following categories: sequence generation, incomplete outcome data, selective outcome reporting, and other biases (e.g., whether or not to specify the method for measuring CRP/hs-CRP). Discrepancies were resolved by discussions with a third investigator (HX). The quality of evidence for each outcome in the pairwise meta-analysis and significant results in the network meta-analyses were evaluated based on the GRADE (Grading of Recommendations Assessment, Development, and Evaluation) process.

### Statistical analysis

The outcome was plasma CRP/hs-CRP level at the final measured point. Data were standardized to mean and standard deviation (*SD*) (26), and units of CRP were converted to mg/L. Placebo and no-interventions were combined as the control groups to provide more evidence for comparisons among statins. We presented the pooled results as weighted mean differences (WMD) and 95% confidence intervals (CI) (16, 27). *P*-values of <0.05 were considered significant.

We performed a pairwise meta-analysis to compare the efficacy of statins and control on serum CRP levels using the Review Manager 5.4 software (The Cochrane Collaboration, Software Update, Oxford, UK). Multi-arm studies were split into comparisons between statins and controls, and the number of participants in the control group was proportionally divided into a new control group to ensure that the total number of participants was exact (25, 28). Due to the high heterogeneity of included studies, we chose a random-effects model to estimate the overall effect, which might provide more conservative results (29). For sensitivity analyses, the robustness of the pooled results was tested by leave-one-out influence analysis.

For NMA, we produced an evidence network plot using the Stata 16 software (STATA Corporation, College Station, TX, USA). The aggregate data drug information system ADDIS 1.16.5 software (Drug Information System, Groningen, The Netherlands) was used to generate the Bayesian random-effects model. We generated a Markov Chain Monte Carlo (MCMC) model to incorporate the efficacy of direct and indirect comparisons and rank the interventions with ranking probabilities (30, 31). The convergence of the MCMC model was assessed using the Brooks-Gelman-Rubin method, which compares within-chain and between-chain variance to calculate the potential scale reduction factor (PSRF) (32). The closer PSRF approaches 1, the better the convergence. Typically, an acceptable PSRF is <1.05 (33). To evaluate the inconsistency of NMA, we used a node-split model to assess the consistency of direct and indirect comparisons. The consistency model was adopted if the *P*-value of >0.05; otherwise, the inconsistent model was used (34).

CRP and hs-CRP have different measurement accuracies, and some studies did not mention the actual measurement clearly; moreover, participants with different diseases, especially those with acute coronary syndromes (ACS), might have higher inflammation levels (35). Therefore, we performed subgroup analyses for CRP measurement (including CRP, hs-CRP, and CRP/hs-CRP with a clear measurement method), population (including CHD, dyslipidemia, ACS, and non-ACS), and treatment duration (less than or more than 12 months). If a study included a population with ACS and dyslipidemia, it would belong to the CHD and ACS subgroups. If there were inconsistencies in the evidence, conclusions were treated cautiously.

## Results

### Eligible studies, risks of bias assessment, and quality of evidence

**Figure 1:**
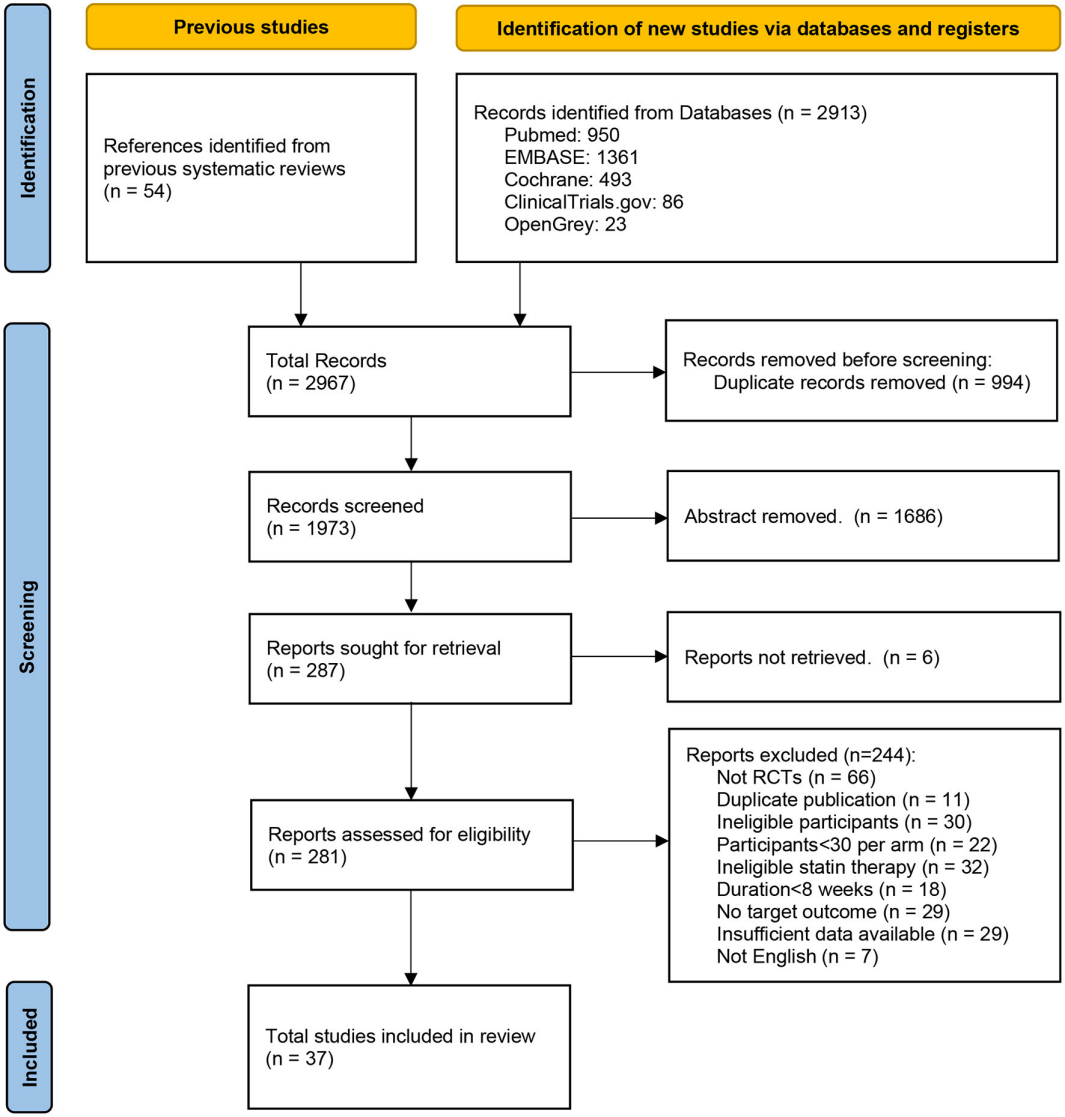
Flow diagram of the study selection progress.

According to the search strategy, we retrieved 2,804 potential eligible papers from the three databases. After screening, 37 studies (19–23, 36–69) with 17,410 participants and 20 interventions were included (Figure 1). Among them, nine studies directly compared a statin with control, 22 compared two different types or doses of statins, and six were multi-arm trials that performed a comparison between at least two different types or dosages of statins and control. The RCTs included five statins (atorvastatin, rosuvastatin, pitavastatin, simvastatin, and pravastatin) at varying dosages. Table 1 shows the baseline characteristics of each included study.

**Table 1:** Baseline characteristics of the included studies.

**Study ID**	**Location**	**Disease**	**Sample size (** ***n*** **)**	**Mean age (year)**	**Male sex (%)**	**Intervention** **(per day)**	**Course**	**Other intervention**
Allen, 2002 (36) (ARBITER)	USA	hypercholesterolemia	161	59.53	71.46	Atorvastatin 80 mg vs. Pravastatin 40 mg	12 m	Unclear
Andrew, 2015 (37) (LIPID)	Australia and New Zealand	CHD (history of ACS)	7,863	60.9	82.5	Pravastatin 40 mg vs. Placebo	12 m	Routine therapy
Cheuk-Man, 2007 (38)	unclear	CHD and hypercholesterolemia	112	66	81.81	Atorvastatin 10 mg vs. Atorvastatin 80 mg	26 w	Unclear
Dan, 2017 (39)	China	NSTE-ACS	83	60.55	73.5	Rosuvastatin 10 mg vs. Rosuvastatin 20 mg	12 w	NSTE-ACS standard therapy (including aspirin, clopidogrel, β-blockers, and angiotensin-converting enzyme inhibitors/ angiotensin II receptor antagonists)
Guo, 2017 (40)	China	ACS	137	60.59	50.58	Rosuvastatin 10 mg vs. Rosuvastatin 20 mg vs. Blank	12 w	PCI; routine therapy
Haiyan, 2009 (41)	China	hypercholesterolemia	69	58.46	52.18	Atorvastatin 10 mg vs. Rosuvastatin 10 mg	12 w	Unclear
Haralampos, 2004 (23)	Greece	Dyslipidemia	180	58.3	61.1	Atorvastatin 40 mg vs. Simvastatin 40 mg	3 m	National Cholesterol Education Program diet
Hiro, 2009 (42) (JAPAN-ACS)	Japan	ACS and hypercholesterolemia (The patients were enrolled within 72 h after PCI.)	307	62.45	81.74	Atorvastatin 20 mg vs. Pitavastatin 4 mg	8–12 m	ACS standard treatment
Jung Wook, 2019 (43)	Republic of Korea	NSTE-ACS and T2DM	72	64.1	69.16	Pitavastatin 1 mg vs. Pitavastatin 4 mg	12 m	Not mentioned
Komukai, 2014 (44) (EASY-FIT)	Japan	ACS (UA) and untreated dyslipidemia	60	65.45	79.97	Atorvastatin 5mg vs. Atorvastatin 20 mg	12 m	Unclear
Kuei Chuan, 2008 (45)	China	CHD	60	64.95	71.48	Atorvastatin 10 mg vs. Blank	6 m	PCI; diet control; sulfonylurea if necessary
Kwang Kon, 2004 (46)	Korea	CHD	63	31.49	40.6	Simvastatin 20 mg vs. Placebo	14 w	American Heart Association Step I Diet; aspirin; β-blocker therapy
Kwang Kon, 2008 (47)	Korea	hypercholesterolemia	160	58.61	46.82	Simvastatin 10 mg vs. Simvastatin 20 mg vs. Simvastatin 40 mg vs. Simvastatin 80 mg vs. Placebo	2 m	Low-fat diet
Kwang Kon, 2010 (48)	Korea	Hyperlipidemia	138	58.68	44.74	Simvastatin 20 mg vs. Simvastatin 40 mg vs. Placebo	2 m	Unclear
Kwang Kon, 2015 (49)	Korea	hypercholesterolemia	102	57	52.9	Simvastatin 20 mg vs. Placebo	2 m	Unclear
Kwang Kon, 2016 (50)	Korea	hypercholesterolemia	190	57.01	50	Rosuvastatin 5 mg vs. Rosuvastatin 10 mg vs. Rosuvastatin 20 mg vs. Placebo	2 m	Low-fat diet
Mehmet, 2006 (51)	Turkey	ACS	122	57.58	88.13	Atorvastatin 40 mg vs. Blank	6 m	AMI standard treatment (STE-MI: thrombolytic drug; NSTE-MI: clopidogrel, aspirin, heparin, nitrate, β-blocker, and angiotensin- converting enzyme inhibitor)
Moutzouri, 2011 (21)	Greece	Hypercholesterolemia and insulin resistance	100	55.3	34.83	Rosuvastatin 10 mg vs. Simvastatin 40 mg	12 w	Unclear
Nakagomi, 2015 (52)	Japan	Dyslipidemia	153	66	50	Atorvastatin 5 mg vs. Pitavastatin 1 mg	12 m	Unclear
Naohisa, 2015–Kazuo, 2017 (53) (J-STARS)	Japan	Hyperlipidemia and ischemic stroke	1095	66.35	68.95	Pravastatin 10 mg vs. Blank	2 m	Diet and exercise therapies
Qianqian, 2017 (54)	China	CHD (including ACS and SAP)	203	61.38	69.15	Atorvastatin 20 mg vs. Atorvastatin 40 mg	12 w	Unclear
Rehab, 2021 (55)	Egypt	Dyslipidemia and T2DM	197	54.92	46.25	Atorvastatin 40 mg vs. Rosuvastatin 10 mg	6 m	Oral hypoglycemic agents
Robert, 2011 (56)	Poland	Mixed Dyslipidemia and T2DM	96	53	57.46	Simvastatin 40 mg vs. Placebo	90 d	Unclear
Robert, 2011(2) (57)	Poland	Hypercholesterolemia	65	52.87	60.52	Simvastatin 40 mg vs. Placebo	90 d	Diet and exercise counseling
Schwartz, 2001-Kinlay et al. (58) (MIRACL)	122 centers in Europe, North America, South Africa, and Australasia.	ACS	2,402	64	66.01	Atorvastatin 80 mg vs. Placebo	16 w	Unclear
Seung-Jung, 2016 (59) (STABLE)	Korea	CHD	225	62.34	72.94	Rosuvastatin 10 mg vs. Rosuvastatin 40 mg	12 m	Unclear
Shigemasa, 2015 (60)	Japan	Hypercholesterolemia	108	59.85	62.52	Atorvastatin 10 mg vs. Pitavastatin 2 mg	6 m	Unclear
Smilde, 2001 - Sanne, 2002 (ASAP) (20)	Netherlands	Heterozygous familial hypercholesterolemia	268	47.99	39.51	Atorvastatin 80 mg vs. Simvastatin 40 mg	24 m	Unclear
Stephen, 2011 (61)	USA	Dyslipidemia	159	57.98	40.91	Atorvastatin 20 mg vs. Rosuvastatin 10 mg vs. Simvastatin 40 mg vs. Placebo	12 w	Unclear
Stephen, 2011 (62) (SATURN)	USA, Australia, France, German,	CHD	1039	57.65	73.65	Atorvastatin 80 mg vs. Rosuvastatin 40 mg	24 m	Unclear
Steven, 2004 (63) (REVERSAL)	USA	CHD	502	56.2	71.99	Atorvastatin 80 mg vs. Pravastatin 40 mg	18 m	Unclear
Suxia, 2012 (11)	China	CHD	244	60.49	86.84	Atorvastatin 10 mg vs. Atorvastatin 20 mg vs. Atorvastatin 40 mg vs. Atorvastatin 80 mg vs. Placebo	3–6 m	Aspirin (100 mg/day)
Tsuyoshi, 2012–Tsuyoshi, 2013 (64) (TRUTH)	Japan	CHD	101	66.5	83.06	Pitavastatin 4 mg vs. Pravastatin 20 mg	8 m	Unclear
Xin, 2013 (65)	China	ACS (UA)	100	65	60	Atorvastatin 20 mg vs. Atorvastatin 80 mg	9 m	Aspirin (100 mg/day)
Young Joon, 2011 (22)	Korea	CHD	128	58.51	74.02	Atorvastatin 40 mg vs. Rosuvastatin 20 mg	11 m	Unclear
Zamani, 2014 (66)	Iran	ACS	180	59.09	64.49	Atorvastatin 20 mg vs. Atorvastatin 40 mg	3 m	Unclear
Zhuo, 2009 (67)	China	ACS (UA)	166	71	65.1	Atorvastatin 20 mg vs. Atorvastatin 80 mg	8 w	UA routine therapy

*ACS, acute coronary syndrome; CHD, coronary heart disease; NSTE-ACS, non-ST segment elevation acute coronary syndrome; UA, unstable angina pectoris; NSTE-MI, non-ST segment elevation myocardial infarction; STE-MI, ST-segment elevation myocardial infarction; SAP, stable angina pectoris; T2DM, type 2 diabetes mellitus; m, month; w, week; d, days*.

**Figure 2:**
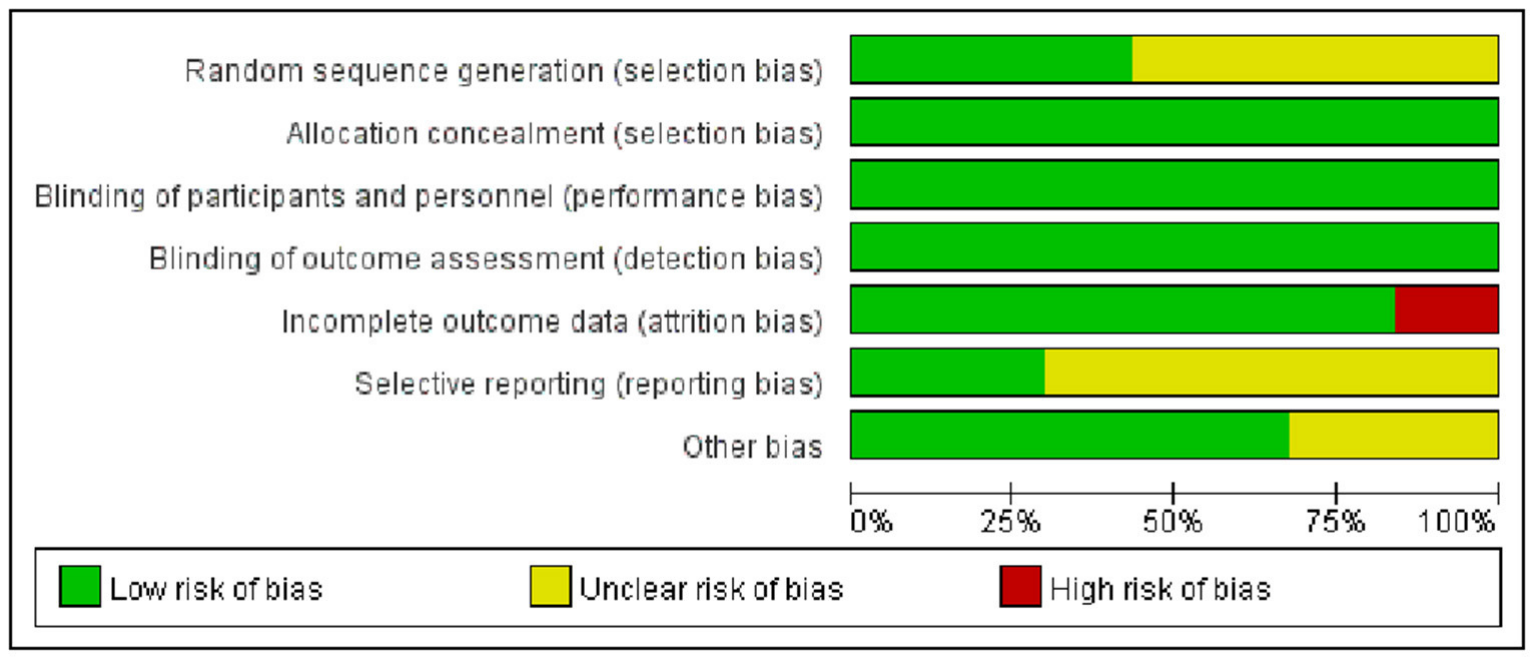
Risk of bias graph of the included studies.

The assessments for bias risk are summarized in Figure 2. All 37 trials reported random assignment, while only 16 studies (20, 21, 36, 38, 39, 42, 49, 52–54, 60–64, 67) explicitly mentioned appropriate random sequence generation methods. Because CRP is an objective measure, the risk of allocation concealment and blinding was low for all studies (25). Concerning incomplete outcome data, five trials (40, 43, 45, 51, 66) did not report the number or reason of loss to follow-up; one trial (65) had a loss to follow-up rate higher than 35%. Regarding selective outcome reporting, 11 studies (19, 39, 42–44, 53, 55, 58, 59, 61, 64) published the protocols and reported the complete results, whereas others were unclear. In addition, 25 studies (20–23, 36–38, 40, 41, 43, 45–52, 54–58, 60, 61) described the detailed methods of measuring CRP/hs-CRP, while the remaining 12 (19, 39, 42, 44, 53, 59, 62–67) were unclear. In the pairwise meta-analysis, the quality of evidence for comparison between statins and non-statin controls was rated as high. In network meta-analyses, the quality of evidence for significant results was rated as high or moderate (Supplementary Table 2).

### Pairwise meta-analysis

**Figure 3:**
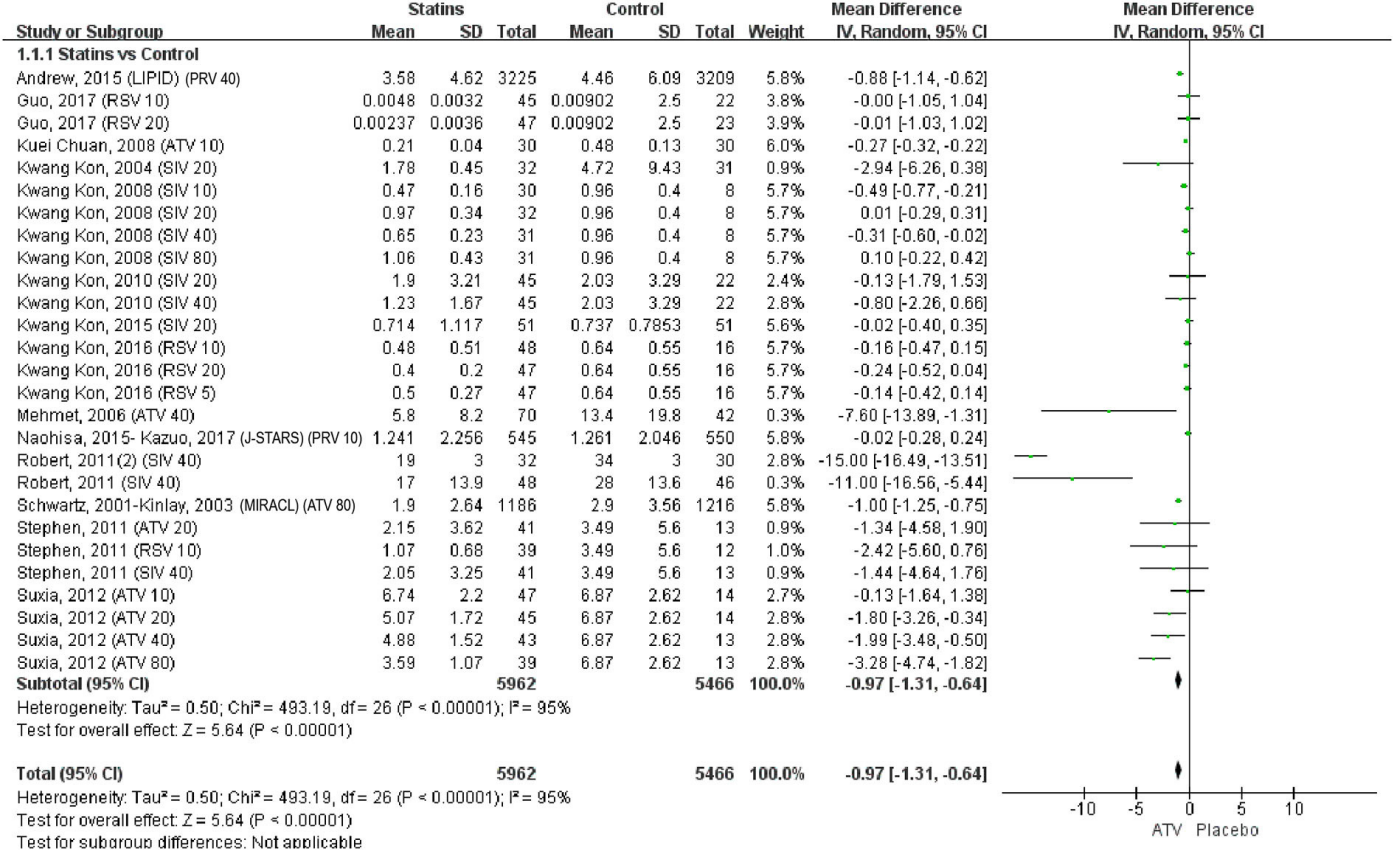
Forest plot of pairwise meta-analysis between statins and control. Note: PRV 40: Pravastatin 40 mg/d; RSV 10: Rosuvastatin 10 mg/d; Rosuvastatin 20 mg/d; ATV 10: Atorvastatin 10 mg/d; SIV 20: Simvastatin 20 mg/d; SIV 10: Simvastatin 10 mg/d; SIV 40: Simvastatin 40 mg/d; SIV 80: Simvastatin 80 mg/d; RSV 5: Rosuvastatin 5 mg/d; ATV 40: Atorvastatin 40 mg/d; PRV 10: Pravastatin 10 mg/d; ATV 80: Atorvastatin 80 mg/d; ATV 20: Atorvastatin 20 mg/d.

The pairwise meta-analysis compared the effects of statins and control on serum CRP levels. As shown in Figure 3, compared with control, statins significantly reduced CRP levels (WMD = −0.97, 95% CI [−1.31, −0.64], *P* < 0.0001, *I*^2^ = 95%). The leave-one-out influence analyses showed that the associations between statins and CRP levels were not determined by any individual study (Supplementary Table 3).

### Network meta-analysis

#### Network evidence

**Figure 4:**
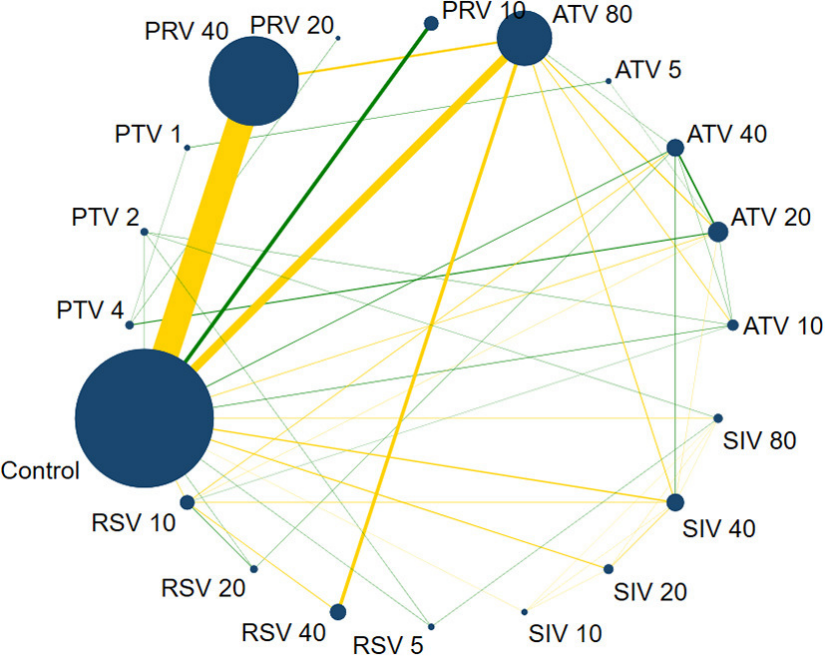
Network evidence plot on CRP. The size of nodes is directly proportional to the number of studies. The lines link two direct-comparison interventions, their thickness is proportional to the number of comparisons, and the green links represent at least one double-blind comparison. PTV 4: Pitavastatin 4 mg/d; PTV 2: Pitavastatin 2 mg/d; PTV 1: Pitavastatin 1 mg/d; PRV 40: Pravastatin 40 mg/d; PRV 20: Pravastatin 20 mg/d; PRV 10: Pravastatin 10 mg/d; ATV 80: Atorvastatin 80 mg/d; ATV 5: Atorvastatin 5 mg/d; ATV 40: Atorvastatin 40 mg/d; ATV 20: Atorvastatin 20 mg/d; ATV 10: Atorvastatin 10 mg/d; SIV 80: Simvastatin 80 mg/d; SIV 40: Simvastatin 40 mg/d; SIV 20: Simvastatin 20 mg/d; SIV 10: Simvastatin 10 mg/d; RSV 5: Rosuvastatin 5 mg/d; RSV 40: Rosuvastatin 40 mg/d; RSV 20: Rosuvastatin 20 mg/d; RSV 10: Rosuvastatin 10 mg/d.

Figure 4 shows the network evidence of 19 statin therapies and control (placebo and no intervention). As shown in Figure 4, control was the most used intermediary comparator. The most common comparisons occurred between pravastatin 40 mg/day and control, followed by atorvastatin 80 mg/day vs. control.

#### NMA of statins on CRP

All 37 studies were included in the NMA (Supplementary Table 4). Overall, only simvastatin 40 mg/day (WMD = −4.07, 95% CI= [−6.52, −1.77]) and atorvastatin 80 mg/day (WMD = −3.32, 95% CI= [−6.02, −0.83]) were significantly better than control among 19 statin therapies. We performed the rank-possibility of statins on lowering CRP. Rank 1 was the worst, and rank 20 was the best (70). Supplementary Figure 1A shows that simvastatin 40 mg/day has the highest *P*-value of rank 20; therefore, it also indicates that simvastatin 40 mg/day might be the best method for lowering CRP (rank *P* = 0.18).

We performed a subgroup analysis based on the measurement method of CRP, including CRP, hs-CRP, and CRP/hs-CRP with a clear measurement method (Supplementary Table 4). The CRP subgroup contained three studies with three interventions and 1,679 participants. We performed both consistency model and inconsistency model because there were no closed loops to conduct node-splitting analysis for assessing inconsistency. Atorvastatin 80 mg/day might be the best at lowering CRP levels (rank *P* = 0.79) among atorvastatin 80 mg/day, pravastatin 40 mg/day, and rosuvastatin 40 mg/day (Supplementary Figure 1B). Moreover, atorvastatin 80 mg/day was significantly better than pravastatin 40 mg/day in both the consistency model (WMD = −1.23, 95% CI = [−2.48, −0.08]) and the inconsistency model (WMD = −1.25, 95% CI= [−2.53, −0.08]). However, owing to the limited studies, the results should be interpreted with caution. The hs-CRP subgroup included 33 studies with 20 interventions. According to the ranking possibility, the best statins for reducing CRP might be pravastatin 40 mg/day (rank *P* = 0.15), simvastatin 40 mg/day (rank *P* = 0.12), or rosuvastatin 40 mg/day (rank *P* = 0.10) (Supplementary Figure 1C). Nevertheless, only simvastatin 40 mg/day (WMD = −4.10, 95% CI= [−6.83, −1.60]) and atorvastatin 80 mg/day (WMD = −3.66, 95% CI = [−7.01, −0.58]) were significantly better than control. Comprehensive considering the ranking and *P-*value, simvastatin 40 mg/day might be the best therapy for lowering CRP levels. The subgroup of CRP/hs-CRP with a clear measurement method contained 23 studies with 14 interventions. Among them, simvastatin 40 mg/day appeared to be the best (rank *P* = 0.20) (Supplementary Figure 1D). Furthermore, only simvastatin 40 mg/day showed a statistically significant difference compared to control (WMD = −4.28, 95% CI = [−7.21, −1.43]).

We conducted another subgroup analysis in terms of population, including CHD, dyslipidemia, ACS, and non-ACS subgroups. The CHD subgroup included 21 studies with 14 interventions. However, there are two inconsistent comparisons; thus, we used the inconsistency model (Supplementary Table 4), which should be interpreted cautiously. In total, 14 studies were included in the dyslipidemia subgroup. The results showed that pitavastatin 2 mg/day tends to be the best (rank *P* = 0.18) (Supplementary Figure 1E); however, there were no significant differences among the 15 interventions (*P* > 0.05). The ACS subgroup included 11 studies. Compared with other interventions, atorvastatin 80 mg/day might be the most effective strategy to reduce the CRP levels (rank *P* = 0.30). However, the comparisons among the nine interventions also showed no significant difference (*P* > 0.05) (Supplementary Figure 1F). Although 29 studies met the criteria of the non-ACS subgroup, only 27 studies were available for indirect comparisons. Simvastatin 40 mg/day has the highest probability of being the best for reducing CRP levels (rank *P* = 0.21) (Supplementary Figure 1G). Furthermore, simvastatin 40 mg/day (WMD = −4.34, 95% CI= [−7.10, −1.76]) was also significantly better than control among 16 interventions (*P* < 0.05).

We conducted the third subgroup analysis according to the treatment duration. For the <12-month duration subgroup, simvastatin 40 mg/day (rank *P* = 0.21) appeared to be the best strategy (Supplementary Figure 1H). Moreover, simvastatin 40 mg/day (WMD = −4.29, 95% CI = [−7.18, −1.55]) and atorvastatin 80 mg/day (WMD = −3.66, 95% CI = [−7.37, −0.19]) were significantly better than control for reducing CRP levels. Although 9 studies were eligible for the ≥12-month duration subgroup, only six studies were available for NMA analysis. Given that there were no closed loops to assess inconsistency, we conducted consistency and inconsistency models. In the consistency model, atorvastatin 80 mg/day (rank *P* = 0.40) and simvastatin 40 mg/day (rank *P* = 0.36) were most likely to be the best for reducing CRP levels (Supplementary Figure 1I). Atorvastatin 80 mg/day was significantly better than pravastatin 40 mg/day (WMD = −1.27, 95% CI = [−2.56, −0.11]) and control (WMD = −2.13, 95% CI = [−4.24, −0.13]) in the consistency and inconsistency models (Supplementary Table 4).

### Consistency and convergence analysis

We performed node-splitting analysis to evaluate inconsistency by comparing direct and indirect effects (Supplementary Table 5). In ten comparisons, seven showed no significant inconsistency, suggesting that the consistency model is reliable. The CRP and ≥12-month duration subgroups could not form closed loops to conduct node-splitting analysis; therefore, we conducted both consistency and inconsistency models; the results of the two models were consistent, suggesting that the results are reliable. The CHD subgroup had two inconsistent comparisons between direct effect and indirect effect (*P* < 0.05); therefore, we generated an inconsistency model; however, the results of these two subgroups should be interpreted with caution (34, 71). In addition, the PSRF was between 1.00 and 1.05, indicating that the analysis had good convergence (70).

## Discussion

To the best of our knowledge, this is the first study to compare the effects of different types and doses of statins on plasma CRP levels in patients with dyslipidemia or CHD.

The pairwise meta-analysis showed that, compared with control, statins decreased CRP levels, which was consistent with previous studies. The JUPITER (Justification for the Use of Statins in Prevention: an Intervention Trial Evaluating Rosuvastatin) was an RCT investigating the anti-inflammatory effects of rosuvastatin in apparently healthy people with elevated hs-CRP levels (72). It showed that rosuvastatin reduced LDL-C levels by 50% and hs-CRP levels by 37%; rosuvastatin significantly reduced the occurrence of major adverse cardiovascular events. Similarly, the HOPE-3 (Heart Outcomes Prevention Evaluation-3) study on intermediate-risk participants without cardiovascular disease supported the hs-CRP-lowering effect of rosuvastatin regardless of CRP and lipid levels at baseline (73). Other systematic reviews supported the role of statins in reducing hs-CRP in patients with cardiovascular diseases (74), stroke (75), and apparently healthy people or patients with chronic diseases (76). Inconsistently, a meta-analysis (77) showed no significant difference between statins and control in lowering the hs-CRP level in atherosclerosis (WMD = −1.61, *P* = 0.09); however, this subgroup only included three trials with 236 participants, which was too few to obtain reliable results.

Dyslipidemia and inflammation are closely interconnected drivers of atherosclerotic heart disease (78). Correspondingly, statins are pleiotropic drugs that lower serum cholesterol by inhibiting hepatic cholesterol biosynthesis and exert cardiovascular protective effects such as anti-inflammation (10). It remains inconclusive whether the anti-inflammatory effects of statins are independent of their lipid-lowering efficacy. Labos et al. used Egger regression to reanalyze the available previous RCT data of statins (79). The study showed that each 1 mmol/L change in LDL-C with statin therapy was associated with a hazard ratio of 0.77 in cardiovascular endpoints with an intercept indistinguishable from zero, suggesting that statins' cardiovascular benefits were entirely derived from LDL-C lowering. Fernando et al. suggested that this analysis should use multivariable (and not “standard”) Egger regression (80). In contrast, in the Cholesterol and Recurrent Events (CARE) trial that investigated inflammation and coronary events after myocardial infarction, statins reduced CRP levels independently of LDL-C (81). Subsequently, a *post-hoc* analysis of the Air Force/Texas Coronary Atherosclerosis Prevention Study (AFCAPS/TexCAPS) reported that compared to individuals with low levels of LDL-C and CRP, those with low LDL-C but elevated CRP levels benefited markedly from lovastatin, suggesting anti-inflammatory activity independent of lipid-lowering (82). The Pravastatin Inflammation/CRP Evaluation (PRINCE) trial demonstrated that pravastatin 40 mg/day significantly reduced plasma CRP levels independent of any changes in LDL levels (83). Unlike clinical studies with inconsistent conclusions, experimental studies assessed the anti-inflammatory effects of statins independent of their lipid-lowering action (17).

C-reactive protein is considered a nonspecific marker of inflammation, produced in response to the action of IL-6, IL-1, or TNF-α (17). It remains unclear whether patients would benefit if CRP were a therapeutic target, although CRP has attracted attention for its applications in screening and risk stratification (84–86). Nevertheless, anti-inflammatory therapies have shown compelling effects in preventing cardiovascular events recently. The Canakinumab Anti-inflammatory Thrombosis Outcome Study (CANTOS) demonstrated that canakinumab, an IL-1β blocker (150 mg for every 3 months), reduced the incidence of nonfatal myocardial infarction, nonfatal stroke, and cardiovascular death (6). Colchicine is an anti-inflammatory drug for treating gout, familial Mediterranean fever, and pericarditis. It has shown promising efficacy in atherosclerotic heart disease. The Colchicine Cardiovascular Outcomes Trial (COLCOT) found that 0.5 mg of colchicine daily significantly lowered the risk of ischemic cardiovascular events in patients who suffered a myocardial infarction within 30 days (7). Subsequently, the Low-Dose Colchicine 2 trial (LoDoCo2), an RCT that involved 5,522 patients with chronic coronary disease, revealed that 0.5 mg/day of colchicine significantly lowered the risk of cardiovascular events (8). The evidence of these anti-inflammatory therapies suggests an approach to treating atherosclerotic disease besides lipid-lowering. Recently, a position paper of the European Society of Cardiology stated that, given the strong association among inflammation, lipids, and atherosclerosis, it would be helpful to assess the inflammatory response to lipid-lowering interventions, thereby establishing the optimal dose and type of lipid-lowering therapy for cardiovascular prevention (11).

The results of NMA showed that simvastatin 40 mg/day appeared to be the best for lowering CRP among the included statin therapies. Simvastatin is a lipophilic statin and an inactive prodrug hydrolyzed in the liver to its major active β-hydroxy acid metabolite (87). Compared with other lipid-lowering agents, simvastatin might be superior in reducing the risk of major adverse cardiovascular events in hypertriglyceridemic patients (88). Consistent with our findings, Mitra et al. supported the notion that lipophilic statins (such as simvastatin) at high-intensity dosage could significantly decrease inflammatory factor TNF-α (89). In contrast, Neda et al. tested the orientation of ligands (statins) and phosphorylcholine (the standard ligand of CRP) at the CRP active site using Molecular Operating Environment software (18). The docking experiments showed that rosuvastatin had the most robust interaction with CRP, followed by fluvastatin, pitavastatin, atorvastatin, pravastatin, simvastatin, and lovastatin. However, in addition to directly acting on CRP, statins reduce inflammation *via* ICAM-1 and VCAM-1 (17), and the evidence from *in silico* studies requires experimental studies for support. In terms of dosage, according to the 2013 ACC/AHA Guideline (90), 40 mg/day is the maximum recommended dose of simvastatin because of the risk of rhabdomyolysis at higher doses, although it is classified as moderate-intensity statin therapy. Similar to our results, a higher intensity dosage is more likely to have better anti-inflammatory effects (89). In addition, high-dose statins (e.g., simvastatin 40 mg/day and atorvastatin 80 mg/day) are associated with the most significant benefits of secondary prevention in patients with ischemic stroke or transient ischemic attack (91).

We performed subgroup analyses to determine the heterogeneity. In the hs-CRP, non-ACS, <12-month duration, and clear measurement method subgroups, simvastatin 40 mg/day appeared to be the best strategy for CRP-lowering, consistent with the NMA. In the ≥12-month duration subgroup, atorvastatin 80 mg/day was most likely to be the best. The evidence from the ≥12-month duration subgroup is more clinically meaningful because statins are long-term drugs. In the CRP subgroup, atorvastatin 80 mg/day was most likely to be the best for reducing CRP levels, while there were only three studies, which made the results unpersuasive. Conversely, there were no significant differences in dyslipidemia and ACS subgroups. Previous research showed a significant difference between statins and placebo in ACS (76). This discrepancy might be caused by the limited number and heterogeneity of included trials.

Our study has some advantages. First, we performed an NMA of RCTs, which could compare multiple treatments and enable us to synthesize data with direct and indirect evidence (30). Compared to previous meta-analyses (76, 77), this study could incorporate all available data to assess interventions more accurately (70). Second, the results were highly consistent between the direct meta-analysis and NMA and the NMA and NMA subgroups, suggesting a stable result. Finally, our study provides the ranking possibilities of different statins, which can help clinicians make choices when faced with elevated CRP levels in patients with CHD.

Although we strictly followed the PRISMA extension statement for NMA, there are some limitations. First, it would be more clinically meaningful if we included a subgroup of baseline CRP levels; however, this is challenging because these studies chose participants according to disease rather than CRP level. Second, there was significant heterogeneity among included studies. To resolve the heterogeneity, we used a random-effects model, which may have influenced differences in study design and trial populations, as well as statistical heterogeneity in some of our results (70). In addition, we conducted subgroup analyses in terms of the measurement method of CRP, the population, or the treatment duration. A leave-one-out influence analysis was performed to test the robustness of the pooled results. The results of this study can still be considered credible. Third, owing to the limited number of trials, we could not include all statin therapies recommended by the guidelines (90) and did not differentiate among statins from various brands, which might lead to errors; nevertheless, this study covered statin prescriptions commonly used in clinical, and all statins are approved, commercially available drugs. Fourth, serum CRP levels are influenced by treatment duration, while the traditional meta-analysis and NMA cannot elucidate the changing effects over time (92). We thereby excluded the studies with treatment <8 weeks and performed a subgroup analysis of treatment duration to partly solve the problem. Finally, our study only included patients with dyslipidemia or CHD, whereas statins are used more widely; however, this also reduced the clinical heterogeneity.

## Conclusion

Statins reduce serum CRP levels in patients with dyslipidemia or CHD. Simvastatin 40 mg/day might be the most effective therapy, and atorvastatin 80 mg/day showed the best long-term effect. This study provides a reference for choosing statin therapy based on LDL-C and CRP levels.

## Data availability statement

The original contributions presented in the study are included in the article/Supplementary material, further inquiries can be directed to the corresponding authors.

## Author contributions

HX, JQJ, and JZ designed this study. JZ and XW searched the databases and wrote the original draft. WT and TXW screened the publications and extracted data. RL and JDJ specified the data. JZ and LS performed the analysis. XW and HX provided methodological guidance. TW and ZZ normalized the figures and tables. All authors reviewed the manuscript.

## Funding

The study was supported by the Chinese Academy of Chinese Medical Sciences Innovation Fund (CACMS Innovation Fund, CI2021A00917), Chinese Academy of Traditional Chinese Medicine Science and Technology Major Achievement Guidance Project (ZZ13-ZD-03), and Central Public Welfare Research Institutes of China Academy of Chinese Medical Sciences (ZZ13-YQ-017-C1).

## Conflict of interest

All authors declare that the research was conducted in the absence of any commercial or financial relationships that could be construed as a potential conflict of interest.

## Publisher's note

All claims expressed in this article are solely those of the authors and do not necessarily represent those of their affiliated organizations, or those of the publisher, the editors and the reviewers. Any product that may be evaluated in this article, or claim that may be made by its manufacturer, is not guaranteed or endorsed by the publisher.
