# Supplementary material for: The effect of various types and doses of statins on C-reactive protein levels in patients with dyslipidemia or coronary heart disease: A systematic review and network meta-analysis
Source: Front Cardiovasc Med. 2022 Jul 27;9:936817. doi: 10.3389/fcvm.2022.936817 (PMC9363636; doi:10.3389/fcvm.2022.936817)
Supplement: Supplementary file 2 [file Table_2.docx]

**Supplementary Table 2. GRADE profile for pairwise meta-analysis and significant results from network meta-analyses.**

| **Pairwise meta-analysis** | | | | | | | | | | | | | |
| --- | --- | --- | --- | --- | --- | --- | --- | --- | --- | --- | --- | --- | --- |
| No of participants,  No of studies | | Risk of bias | | Inconsistency | | Indirectness | | Imprecision | | Publication bias | | Overall certainty of evidence | |
| 17410, 37 | | not serious | | not serious | | not serious | | not serious | | none | | ⨁⨁⨁⨁ High | |
| **Significant results from network meta-analyses** | | | | | | | | | | | | | |
| Treatment | Comparator | | Direct Evidence | | | | Indirect Evidence | | | | Combined Evidence | | |
|  |  |  | OR (95% CI) | | Quality | | OR (95% CI) | | Quality | | OR (95% CI) | | Quality |
| Simvastatin 40mg | Control | | -5.04 (-7.81, -2.33) | | Moderate* | | -2.16 (-6.89, 2.23) | | Moderate* | | -4.07 (-6.52, -1.77) | | Moderate* |
| Control | Atorvastatin 80mg | | 2.18 (-2.07, 6.28) | | High | | 4.11 (1.02, 7.37) | | High | | 3.32 (0.83, 6.02) | | High |

OR: odds ratio, CI: confidence interval

* Imprecision due to small sample size for direct comparison or for certain treatment comparisons that were involved in the indirect evidence.
