# Supplementary material for: The effect of various types and doses of statins on C-reactive protein levels in patients with dyslipidemia or coronary heart disease: A systematic review and network meta-analysis
Source: Front Cardiovasc Med. 2022 Jul 27;9:936817. doi: 10.3389/fcvm.2022.936817 (PMC9363636; doi:10.3389/fcvm.2022.936817)
Supplement: Supplementary file 3 [file Table_3.docx]

**Supplementary Table 3. The leave-one-out influence analysis.**

| **Study** | **Mean Difference [95%CI]** |
| --- | --- |
| Omitting Andrew, 2015 (LIPID) (PRV 40) | -1.00 [-1.35, -0.64] |
| Omitting Guo, 2017 (RSV 10) | -1.02 [-1.36, -0.67] |
| Omitting Guo, 2017 (RSV 20) | -1.02 [-1.36, -0.67] |
| Omitting Kuei Chuan, 2008 (ATV 10) | -1.20 [-1.65, -0.75] |
| Omitting Kwang Kon, 2004 (SIV 20) | -0.96 [-1.30, -0.62] |
| Omitting Kwang Kon, 2008 (SIV 10) | -1.03 [-1.39, -0.67] |
| Omitting Kwang Kon, 2008 (SIV 20) | -1.05 [-1.41, -0.70] |
| Omitting Kwang Kon, 2008 (SIV 40) | -1.04 [-1.40, -0.68] |
| Omitting Kwang Kon, 2008 (SIV 80) | -1.06 [-1.41, -0.70] |
| Omitting Kwang Kon, 2010 (SIV 20) | -1.00 [-1.34, -0.65] |
| Omitting Kwang Kon, 2010 (SIV 40) | -0.98 [-1.33, -0.64] |
| Omitting Kwang Kon, 2015 (SIV 20) | -1.04 [-1.40, -0.69] |
| Omitting Kwang Kon, 2016 (RSV 10) | -1.04 [-1.40, -0.69] |
| Omitting Kwang Kon, 2016 (RSV 20) | -1.05 [-1.41, -0.68] |
| Omitting Kwang Kon, 2016 (RSV 5) | -1.05 [-1.41, -0.69] |
| Omitting Mehmet, 2006 (ATV 40) | -0.95 [-1.29, -0.62] |
| Omitting Naohisa, 2015- Kazuo, 2017 (J-STARS) (PRV 10) | -1.06 [-1.42, -0.70] |
| Omitting Robert, 2011 (SIV 40) | -0.93 [-1.27, -0.60] |
| Omitting Robert, 2011(2) (SIV 40) | -0.40 [-0.59, -0.22] |
| Omitting Schwartz, 2001-Kinlay, 2003 (MIRACL) (ATV 80) | -0.99 [-1.34, -0.63] |
| Omitting Stephen, 2011 (ATV 20) | -0.97 [-1.31, -0.63] |
| Omitting Stephen, 2011 (RSV 10) | -0.96 [-1.30, -0.62] |
| Omitting Stephen, 2011 (SIV 40) | -0.97 [-1.31, -0.63] |
| Omitting Suxia, 2012 (ATV 10) | -1.00 [-1.34, -0.66] |
| Omitting Suxia, 2012 (ATV 20) | -0.95 [-1.29, -0.61] |
| Omitting Suxia, 2012 (ATV 40) | -0.94 [-1.29, -0.60] |
| Omitting Suxia, 2012 (ATV 80) | -0.90 [-1.24, -0.56] |

PRV 40: Pravastatin 40 mg/d; RSV 10: Rosuvastatin 10 mg/d; Rosuvastatin 20 mg/d; ATV 10: Atorvastatin 10 mg/d; SIV 20: Simvastatin 20 mg/d; SIV 10: Simvastatin 10 mg/d; SIV 40: Simvastatin 40 mg/d; SIV 80: Simvastatin 80 mg/d; RSV 5: Rosuvastatin 5 mg/d; ATV 40: Atorvastatin 40 mg/d; PRV 10: Pravastatin 10 mg/d; ATV 80: Atorvastatin 80 mg/d; ATV 20: Atorvastatin 20 mg/d.
