# Supplementary material for: The effect of various types and doses of statins on C-reactive protein levels in patients with dyslipidemia or coronary heart disease: A systematic review and network meta-analysis
Source: Front Cardiovasc Med. 2022 Jul 27;9:936817. doi: 10.3389/fcvm.2022.936817 (PMC9363636; doi:10.3389/fcvm.2022.936817)
Supplement: Supplementary file 4 [file Table_4.docx]

**Supplementary Table 4. The NMA results for various types and dosages of statins.**

| **All included interventions** | | | | | | | | | | | | | | | | | | | |
| --- | --- | --- | --- | --- | --- | --- | --- | --- | --- | --- | --- | --- | --- | --- | --- | --- | --- | --- | --- |
| ATV 10 |  |  |  |  |  |  |  |  |  |  |  |  |  |  |  |  |  |  |  |
| 0.35 (-3.28, 3.84) | ATV 20 |  |  |  |  |  |  |  |  |  |  |  |  |  |  |  |  |  |  |
| 1.22 (-2.51, 4.92) | 0.85 (-2.02, 3.76) | ATV 40 |  |  |  |  |  |  |  |  |  |  |  |  |  |  |  |  |  |
| 0.91 (-5.66, 7.35) | 0.54 (-4.86, 5.88) | -0.31 (-6.53, 5.77) | ATV 5 |  |  |  |  |  |  |  |  |  |  |  |  |  |  |  |  |
| 2.49 (-0.66, 5.74) | 2.13 (-0.68, 5.13) | 1.30 (-1.96, 4.65) | 1.62 (-4.59, 7.89) | ATV 80 |  |  |  |  |  |  |  |  |  |  |  |  |  |  |  |
| -0.84 (-3.98, 2.25) | -1.21 (-4.06, 1.72) | -2.05 (-5.18, 1.06) | -1.74 (-7.84, 4.54) | -3.32 (-6.02, -0.83) | Control |  |  |  |  |  |  |  |  |  |  |  |  |  |  |
| -0.80 (-7.50, 5.68) | -1.17 (-7.85, 5.25) | -1.99 (-8.71, 4.43) | -1.69 (-10.06, 6.91) | -3.28 (-9.81, 2.81) | 0.04 (-5.89, 5.74) | PRV 10 |  |  |  |  |  |  |  |  |  |  |  |  |  |
| 0.77 (-7.97, 9.83) | 0.42 (-7.42, 8.72) | -0.41 (-8.88, 8.39) | -0.10 (-8.87, 9.10) | -1.73 (-10.26, 7.15) | 1.64 (-6.83, 10.47) | 1.56 (-8.62, 12.13) | PRV 20 |  |  |  |  |  |  |  |  |  |  |  |  |
| 0.79 (-3.73, 5.21) | 0.40 (-3.80, 4.72) | -0.44 (-4.83, 4.09) | -0.09 (-6.95, 6.75) | -1.73 (-5.22, 1.69) | 1.62 (-2.12, 5.41) | 1.57 (-5.27, 8.65) | -0.02 (-9.50, 9.03) | PRV 40 |  |  |  |  |  |  |  |  |  |  |  |
| 0.46 (-6.90, 7.57) | 0.11 (-6.17, 6.06) | -0.75 (-7.84, 6.05) | -0.44 (-6.43, 5.41) | -2.04 (-9.20, 4.73) | 1.31 (-5.78, 7.96) | 1.21 (-7.92, 10.33) | -0.34 (-8.86, 7.73) | -0.31 (-8.07, 7.05) | PTV 1 |  |  |  |  |  |  |  |  |  |  |
| 0.18 (-5.50, 6.04) | -0.20 (-6.86, 6.76) | -1.00 (-7.83, 5.92) | -0.71 (-9.34, 8.30) | -2.31 (-8.85, 4.34) | 1.03 (-5.46, 7.56) | 1.02 (-7.58, 9.92) | -0.61 (-11.26, 9.88) | -0.60 (-7.83, 6.88) | -0.23 (-9.27, 9.14) | PTV 2 |  |  |  |  |  |  |  |  |  |
| 0.76 (-5.60, 7.05) | 0.43 (-4.87, 5.53) | -0.41 (-6.47, 5.55) | -0.12 (-6.43, 6.16) | -1.72 (-7.96, 4.12) | 1.64 (-4.41, 7.52) | 1.61 (-6.60, 10.07) | -0.05 (-6.29, 6.20) | 0.01 (-6.85, 6.62) | 0.34 (-4.78, 5.61) | 0.58 (-8.22, 9.08) | PTV 4 |  |  |  |  |  |  |  |  |
| 0.96 (-2.34, 4.23) | 0.60 (-2.43, 3.74) | -0.24 (-3.45, 2.94) | 0.08 (-6.14, 6.51) | -1.54 (-4.57, 1.44) | 1.82 (-0.63, 4.35) | 1.78 (-4.47, 8.34) | 0.21 (-8.60, 8.65) | 0.17 (-4.16, 4.51) | 0.52 (-6.31, 7.80) | 0.78 (-5.92, 7.29) | 0.20 (-5.81, 6.47) | RSV 10 |  |  |  |  |  |  |  |
| 0.58 (-3.56, 4.68) | 0.23 (-3.66, 4.17) | -0.63 (-4.44, 3.20) | -0.31 (-6.99, 6.48) | -1.95 (-5.74, 1.81) | 1.42 (-1.93, 4.64) | 1.39 (-5.21, 8.16) | -0.23 (-9.52, 8.71) | -0.22 (-5.05, 4.57) | 0.11 (-6.94, 7.64) | 0.37 (-6.70, 7.36) | -0.19 (-6.67, 6.54) | -0.39 (-3.52, 2.63) | RSV 20 |  |  |  |  |  |  |
| 1.56 (-3.49, 6.64) | 1.19 (-3.73, 6.07) | 0.35 (-4.68, 5.37) | 0.64 (-6.59, 7.94) | -0.96 (-5.43, 3.34) | 2.40 (-2.29, 7.00) | 2.32 (-5.11, 9.84) | 0.82 (-8.96, 10.11) | 0.76 (-4.76, 6.27) | 1.06 (-6.82, 9.37) | 1.33 (-6.37, 9.01) | 0.75 (-6.24, 8.00) | 0.57 (-3.80, 4.92) | 1.00 (-4.15, 6.14) | RSV 40 |  |  |  |  |  |
| 0.24 (-5.41, 5.90) | -0.17 (-5.54, 5.54) | -1.00 (-6.48, 4.62) | -0.68 (-8.21, 7.35) | -2.27 (-7.65, 3.09) | 1.07 (-3.85, 6.17) | 1.08 (-6.59, 8.87) | -0.55 (-10.48, 9.18) | -0.60 (-6.70, 5.61) | -0.27 (-8.33, 8.48) | 0.06 (-7.96, 7.95) | -0.52 (-7.97, 7.36) | -0.75 (-5.65, 4.22) | -0.33 (-5.56, 4.84) | -1.34 (-7.94, 5.17) | RSV 5 |  |  |  |  |
| 1.42 (-4.37, 7.13) | 1.07 (-4.54, 6.76) | 0.21 (-5.44, 5.97) | 0.53 (-7.10, 8.63) | -1.07 (-6.48, 4.27) | 2.26 (-2.63, 7.22) | 2.23 (-5.30, 9.99) | 0.61 (-9.54, 10.51) | 0.65 (-5.40, 6.76) | 0.96 (-7.22, 9.65) | 1.26 (-7.01, 9.46) | 0.63 (-6.83, 8.58) | 0.47 (-4.94, 5.90) | 0.88 (-5.00, 6.74) | -0.12 (-6.71, 6.55) | 1.19 (-5.77, 8.06) | SIV 10 |  |  |  |
| 0.75 (-3.37, 4.88) | 0.39 (-3.56, 4.31) | -0.44 (-4.56, 3.59) | -0.17 (-6.92, 6.53) | -1.73 (-5.57, 1.85) | 1.61 (-1.22, 4.44) | 1.55 (-4.90, 8.17) | -0.02 (-9.15, 8.97) | -0.06 (-4.65, 4.60) | 0.31 (-6.91, 7.93) | 0.58 (-6.52, 7.67) | -0.06 (-6.44, 6.73) | -0.20 (-3.87, 3.45) | 0.18 (-4.04, 4.45) | -0.78 (-6.09, 4.61) | 0.51 (-5.19, 6.32) | -0.69 (-5.79, 4.46) | SIV 20 |  |  |
| 3.23 (-0.29, 6.90) | 2.89 (-0.34, 6.25) | 2.03 (-1.48, 5.57) | 2.35 (-3.98, 8.60) | 0.77 (-2.33, 3.71) | 4.07 (1.77, 6.52) | 4.04 (-2.01, 10.48) | 2.49 (-6.50, 11.06) | 2.44 (-1.81, 6.68) | 2.78 (-4.06, 10.23) | 3.07 (-3.66, 9.85) | 2.44 (-3.59, 8.89) | 2.27 (-0.53, 5.11) | 2.66 (-1.00, 6.50) | 1.69 (-3.10, 6.74) | 3.00 (-2.25, 8.35) | 1.78 (-3.19, 6.89) | 2.45 (-0.67, 5.75) | SIV 40 |  |
| 0.85 (-4.86, 6.51) | 0.46 (-5.10, 6.13) | -0.38 (-6.01, 5.25) | -0.04 (-7.74, 7.75) | -1.62 (-7.09, 3.66) | 1.66 (-3.25, 6.52) | 1.66 (-5.82, 9.30) | 0.05 (-9.88, 9.72) | 0.09 (-6.02, 6.05) | 0.37 (-7.63, 9.02) | 0.64 (-7.47, 8.67) | 0.03 (-7.48, 7.70) | -0.10 (-5.51, 5.10) | 0.26 (-5.61, 6.09) | -0.71 (-7.21, 5.92) | 0.60 (-6.35, 7.56) | -0.62 (-6.43, 5.19) | 0.13 (-5.18, 5.09) | -2.37 (-7.39, 2.49) | SIV 80 |
| **Subgroup analysis of CRP** | | | | | | | | | | | | | | | | | | | |
| **Consistency modle** | | |  | **Inconsistency modle** | | |  |  |  |  |  |  |  |  |  |  |  |  |  |
| ATV 80 |  |  |  | ATV 80 |  |  |  |  |  |  |  |  |  |  |  |  |  |  |  |
| -1.23 (-2.48, -0.08) | PRV 40 |  |  | -1.25 (-2.53, -0.08) | PRV 40 |  |  |  |  |  |  |  |  |  |  |  |  |  |  |
| -0.44 (-2.04, 1.17) | 0.78 (-1.17, 2.80) | RSV 40 |  | -0.47 (-2.08, 1.22) | 0.78 (-1.20, 2.96) | RSV 40 |  |  |  |  |  |  |  |  |  |  |  |  |  |
| **Subgroup analysis of hs-CRP** | | | | | | | | | | | | | | | | | | | |
| ATV 10 |  |  |  |  |  |  |  |  |  |  |  |  |  |  |  |  |  |  |  |
| 0.36 (-3.59, 4.36) | ATV 20 |  |  |  |  |  |  |  |  |  |  |  |  |  |  |  |  |  |  |
| 1.20 (-2.82, 5.29) | 0.82 (-2.26, 4.04) | ATV 40 |  |  |  |  |  |  |  |  |  |  |  |  |  |  |  |  |  |
| 0.88 (-6.09, 7.91) | 0.53 (-5.28, 6.42) | -0.27 (-6.95, 6.42) | ATV 5 |  |  |  |  |  |  |  |  |  |  |  |  |  |  |  |  |
| 2.79 (-0.90, 6.61) | 2.43 (-0.77, 5.74) | 1.61 (-2.10, 5.38) | 1.89 (-4.76, 8.74) | ATV 80 |  |  |  |  |  |  |  |  |  |  |  |  |  |  |  |
| -0.89 (-4.37, 2.60) | -1.23 (-4.47, 1.94) | -2.10 (-5.46, 1.20) | -1.79 (-8.53, 4.83) | -3.66 (-7.01, -0.58) | Control |  |  |  |  |  |  |  |  |  |  |  |  |  |  |
| -0.90 (-8.18, 6.52) | -1.25 (-8.33, 5.86) | -2.10 (-9.26, 5.27) | -1.77 (-11.10, 7.51) | -3.73 (-10.82, 3.44) | -0.03 (-6.31, 6.47) | PRV 10 |  |  |  |  |  |  |  |  |  |  |  |  |  |
| 0.92 (-9.03, 10.34) | 0.53 (-8.48, 9.03) | -0.30 (-9.86, 8.72) | -0.03 (-9.81, 9.33) | -1.91 (-11.65, 7.20) | 1.79 (-7.76, 10.90) | 1.83 (-9.72, 12.86) | PRV 20 |  |  |  |  |  |  |  |  |  |  |  |  |
| -0.11 (-7.48, 7.33) | -0.48 (-7.66, 6.85) | -1.27 (-8.49, 5.93) | -1.02 (-10.43, 8.48) | -2.88 (-10.24, 4.30) | 0.78 (-5.75, 7.32) | 0.84 (-8.52, 9.95) | -0.98 (-12.09, 10.79) | PRV 40 |  |  |  |  |  |  |  |  |  |  |  |
| 0.53 (-7.22, 8.36) | 0.19 (-6.62, 7.02) | -0.62 (-8.28, 6.80) | -0.37 (-6.86, 6.11) | -2.27 (-9.92, 5.12) | 1.44 (-5.96, 8.95) | 1.39 (-8.49, 11.27) | -0.32 (-9.14, 8.69) | 0.63 (-9.31, 10.41) |  |  |  |  |  |  |  |  |  |  |  |
| 0.29 (-6.21, 6.75) | -0.12 (-7.42, 7.51) | -0.95 (-8.62, 6.73) | -0.71 (-10.04, 8.99) | -2.55 (-10.02, 4.74) | 1.15 (-6.16, 8.35) | 1.17 (-8.65, 10.63) | -0.67 (-11.78, 11.01) | 0.32 (-9.54, 10.02) | -0.32 (-10.12, 9.78) | PTV 2 |  |  |  |  |  |  |  |  |  |
| 0.82 (-6.12, 7.74) | 0.49 (-5.15, 6.25) | -0.28 (-6.89, 6.13) | -0.02 (-6.98, 6.68) | -1.94 (-8.67, 4.57) | 1.76 (-4.84, 8.25) | 1.80 (-7.55, 10.66) | -0.02 (-6.82, 6.86) | 1.01 (-8.34, 9.94) | 0.32 (-5.49, 6.08) | 0.61 (-8.96, 9.76) | PTV 4 |  |  |  |  |  |  |  |  |
| 0.82 (-2.92, 4.58) | 0.44 (-3.15, 3.93) | -0.39 (-3.78, 3.22) | -0.04 (-7.02, 6.54) | -1.97 (-5.76, 1.70) | 1.68 (-1.17, 4.58) | 1.72 (-5.55, 8.78) | -0.02 (-9.27, 9.58) | 0.92 (-6.23, 8.00) | 0.29 (-7.50, 7.87) | 0.56 (-6.88, 7.83) | -0.08 (-6.77, 6.66) | RSV 10 |  |  |  |  |  |  |  |
| 0.40 (-4.21, 4.99) | 0.05 (-4.31, 4.34) | -0.78 (-5.07, 3.41) | -0.47 (-7.75, 6.60) | -2.36 (-6.89, 2.04) | 1.30 (-2.27, 4.98) | 1.30 (-6.19, 8.74) | -0.48 (-9.99, 9.51) | 0.49 (-6.92, 7.83) | -0.15 (-8.08, 8.01) | 0.18 (-7.63, 7.88) | -0.45 (-7.52, 6.73) | -0.38 (-3.77, 2.98) | RSV 20 |  |  |  |  |  |  |
| 0.80 (-6.80, 8.31) | 0.49 (-7.02, 8.02) | -0.36 (-7.86, 7.02) | -0.02 (-9.66, 9.37) | -1.97 (-9.72, 5.57) | 1.73 (-5.65, 8.81) | 1.73 (-8.05, 11.35) | -0.08 (-11.46, 11.62) | 0.86 (-8.87, 10.70) | 0.30 (-9.82, 10.40) | 0.47 (-9.41, 10.66) | -0.00 (-9.52, 9.41) | 0.02 (-6.57, 6.61) | 0.42 (-7.14, 7.96) | RSV 40 |  |  |  |  |  |
| 0.10 (-6.29, 6.48) | -0.27 (-6.56, 6.01) | -1.09 (-7.32, 5.04) | -0.78 (-9.32, 7.55) | -2.65 (-9.22, 3.60) | 1.01 (-4.58, 6.59) | 1.05 (-7.75, 9.52) | -0.82 (-11.24, 10.06) | 0.17 (-8.40, 8.88) | -0.41 (-9.74, 8.76) | -0.16 (-9.14, 8.91) | -0.82 (-9.30, 7.73) | -0.70 (-6.40, 4.82) | -0.32 (-6.09, 5.49) | -0.72 (-9.45, 7.97) | RSV 5 |  |  |  |  |
| 1.28 (-5.12, 7.85) | 0.94 (-5.47, 7.16) | 0.11 (-6.39, 6.38) | 0.33 (-8.12, 9.01) | -1.46 (-7.83, 4.66) | 2.18 (-3.43, 7.78) | 2.19 (-6.36, 10.73) | 0.43 (-9.88, 11.42) | 1.34 (-7.16, 10.05) | 0.69 (-8.58, 10.18) | 1.07 (-8.04, 10.11) | 0.42 (-8.06, 8.98) | 0.51 (-5.52, 6.62) | 0.91 (-5.60, 7.37) | 0.49 (-8.43, 9.22) | 1.20 (-6.59, 8.99) | SIV 10 |  |  |  |
| 0.46 (-4.44, 5.35) | 0.11 (-4.57, 4.91) | -0.72 (-5.52, 4.12) | -0.45 (-7.93, 7.09) | -2.30 (-7.03, 2.27) | 1.34 (-2.17, 5.01) | 1.37 (-6.08, 8.69) | -0.43 (-9.95, 9.66) | 0.52 (-6.87, 8.19) | -0.10 (-8.40, 8.21) | 0.21 (-7.78, 8.27) | -0.40 (-7.80, 7.11) | -0.34 (-4.72, 4.02) | 0.06 (-5.05, 5.15) | -0.35 (-8.27, 7.65) | 0.32 (-6.33, 6.97) | -0.82 (-6.63, 5.02) | SIV 20 |  |  |
| 3.22 (-0.68, 7.31) | 2.85 (-0.67, 6.65) | 2.03 (-1.64, 5.92) | 2.30 (-4.53, 9.42) | 0.43 (-3.07, 3.99) | 4.10 (1.60, 6.83) | 4.14 (-2.75, 11.06) | 2.31 (-6.78, 12.08) | 3.32 (-3.49, 10.43) | 2.66 (-4.96, 10.61) | 2.93 (-4.52, 10.61) | 2.37 (-4.29, 9.29) | 2.40 (-0.82, 5.73) | 2.80 (-1.24, 7.07) | 2.41 (-4.74, 9.99) | 3.11 (-2.77, 9.25) | 1.91 (-3.56, 7.64) | 2.76 (-1.03, 6.79) | SIV 40 |  |
| 0.71 (-5.74, 7.20) | 0.37 (-5.81, 6.75) | -0.48 (-6.63, 5.93) | -0.18 (-8.61, 8.70) | -2.09 (-8.33, 4.00) | 1.60 (-3.73, 7.15) | 1.64 (-6.83, 10.20) | -0.21 (-10.62, 10.98) | 0.79 (-7.54, 9.25) | 0.18 (-8.86, 9.67) | 0.46 (-8.63, 9.81) | -0.14 (-8.53, 8.55) | -0.06 (-6.10, 5.98) | 0.33 (-6.18, 6.76) | -0.15 (-8.96, 8.90) | 0.63 (-7.21, 8.51) | -0.60 (-7.12, 5.92) | 0.25 (-5.38, 6.01) | -2.50 (-8.10, 3.08) | SIV 80 |
| **Subgroup analysis of CRP/hs-CRP with clear measurement method** | | | | | | | | | | | | | | | | | | | |
| ATV 10 |  |  |  |  |  |  |  |  |  |  |  |  |  |  |  |  |  |  |  |
| 1.16 (-5.68, 8.01) | ATV 20 |  |  |  |  |  |  |  |  |  |  |  |  |  |  |  |  |  |  |
| 1.66 (-4.56, 7.99) | 0.48 (-4.96, 5.96) | ATV 40 |  |  |  |  |  |  |  |  |  |  |  |  |  |  |  |  |  |
| 1.61 (-3.58, 6.72) | 0.49 (-6.20, 6.99) | -0.03 (-6.28, 6.01) | ATV 80 |  |  |  |  |  |  |  |  |  |  |  |  |  |  |  |  |
| -1.18 (-5.82, 3.34) | -2.31 (-7.93, 2.92) | -2.81 (-7.71, 1.88) | -2.78 (-7.06, 1.50) | Control |  |  |  |  |  |  |  |  |  |  |  |  |  |  |  |
| -0.10 (-6.85, 6.70) | -1.22 (-9.00, 6.43) | -1.74 (-9.05, 5.42) | -1.69 (-7.34, 3.88) | 1.07 (-4.54, 6.65) | PRV 40 |  |  |  |  |  |  |  |  |  |  |  |  |  |  |
| 0.33 (-7.14, 7.78) | -0.85 (-10.95, 9.19) | -1.32 (-10.84, 8.38) | -1.22 (-10.29, 7.76) | 1.53 (-7.12, 10.35) | 0.45 (-9.76, 10.58) | PTV 2 |  |  |  |  |  |  |  |  |  |  |  |  |  |
| 0.94 (-3.95, 5.76) | -0.20 (-5.64, 5.12) | -0.71 (-5.38, 3.92) | -0.66 (-5.62, 4.30) | 2.09 (-1.03, 5.54) | 1.05 (-5.38, 7.51) | 0.66 (-8.47, 9.37) | RSV 10 |  |  |  |  |  |  |  |  |  |  |  |  |
| 0.22 (-5.78, 6.38) | -0.90 (-7.28, 5.44) | -1.40 (-6.88, 3.95) | -1.40 (-7.31, 4.56) | 1.41 (-3.02, 5.82) | 0.27 (-6.64, 7.41) | -0.07 (-9.44, 9.36) | -0.72 (-5.18, 3.76) | RSV 20 |  |  |  |  |  |  |  |  |  |  |  |
| -0.02 (-7.56, 7.64) | -1.21 (-9.12, 6.78) | -1.69 (-9.12, 5.97) | -1.62 (-9.03, 5.81) | 1.13 (-5.10, 7.64) | 0.08 (-8.40, 8.62) | -0.36 (-10.85, 10.21) | -0.99 (-7.27, 5.43) | -0.33 (-6.79, 6.42) | RSV 5 |  |  |  |  |  |  |  |  |  |  |
| 1.18 (-6.39, 8.88) | 0.02 (-8.13, 8.18) | -0.44 (-8.40, 7.37) | -0.41 (-7.96, 7.12) | 2.38 (-3.81, 8.76) | 1.27 (-6.98, 9.82) | 0.82 (-9.66, 11.65) | 0.27 (-6.83, 7.20) | 0.98 (-6.65, 8.52) | 1.24 (-7.75, 10.15) | SIV 10 |  |  |  |  |  |  |  |  |  |
| 0.51 (-5.44, 6.12) | -0.63 (-6.99, 5.79) | -1.15 (-7.05, 4.56) | -1.13 (-6.46, 4.37) | 1.65 (-1.76, 5.26) | 0.60 (-6.10, 7.30) | 0.15 (-9.14, 9.35) | -0.44 (-5.17, 4.18) | 0.25 (-5.32, 5.97) | 0.59 (-6.88, 7.76) | -0.69 (-7.25, 5.68) | SIV 20 |  |  |  |  |  |  |  |  |
| 3.08 (-1.87, 8.16) | 1.95 (-3.65, 7.54) | 1.49 (-3.78, 6.69) | 1.49 (-2.95, 6.09) | 4.28 (1.43, 7.21) | 3.19 (-2.84, 9.17) | 2.78 (-6.19, 11.79) | 2.16 (-1.55, 5.89) | 2.89 (-1.99, 7.98) | 3.09 (-3.65, 10.01) | 1.93 (-4.56, 8.40) | 2.64 (-1.33, 6.63) | SIV 40 |  |  |  |  |  |  |  |
| 0.56 (-7.27, 7.95) | -0.60 (-8.83, 7.59) | -1.06 (-8.86, 6.55) | -1.04 (-8.45, 6.23) | 1.75 (-4.41, 7.83) | 0.64 (-7.79, 9.12) | 0.26 (-10.37, 10.73) | -0.36 (-7.32, 6.23) | 0.39 (-7.28, 7.85) | 0.61 (-8.25, 9.15) | -0.63 (-8.03, 6.62) | 0.09 (-6.31, 6.50) | -2.54 (-8.86, 3.68) | SIV 80 |  |  |  |  |  |  |
| **Subgroup analysis of CHD** | | | | | | | | | | | | | | | | | | | |
| ATV 10 |  |  |  |  |  |  |  |  |  |  |  |  |  |  |  |  |  |  |  |
| 1.26 (0.06, 2.19) | ATV 20 |  |  |  |  |  |  |  |  |  |  |  |  |  |  |  |  |  |  |
| 1.61 (0.47, 2.68) | 0.36 (-0.31, 1.20) | ATV 40 |  |  |  |  |  |  |  |  |  |  |  |  |  |  |  |  |  |
| 2.22 (-0.67, 5.07) | 0.92 (-1.67, 3.52) | 0.50 (-2.10, 3.36) | ATV 5 |  |  |  |  |  |  |  |  |  |  |  |  |  |  |  |  |
| 2.78 (1.59, 3.57) | 1.46 (0.43, 2.38) | 1.10 (-0.18, 2.11) | 0.57 (-2.29, 3.21) | ATV 80 |  |  |  |  |  |  |  |  |  |  |  |  |  |  |  |
| -0.24 (-0.92, 0.81) | -0.34 (-2.69, 0.80) | -0.71 (-2.98, 0.51) | -1.16 (-5.63, 1.53) | -1.13 (-2.62, -0.43) | Control |  |  |  |  |  |  |  |  |  |  |  |  |  |  |
| 1.43 (-1.37, 4.09) | 0.22 (-2.33, 2.71) | -0.14 (-2.83, 2.40) | -0.72 (-4.16, 3.18) | -1.23 (-3.94, 1.52) | 0.59 (-2.29, 3.80) | PRV 20 |  |  |  |  |  |  |  |  |  |  |  |  |  |
| 1.55 (-0.04, 3.03) | 0.29 (-1.11, 1.67) | -0.07 (-1.66, 1.33) | -0.62 (-3.68, 2.21) | -1.13 (-2.18, -0.03) | 0.89 (-0.11, 1.90) | 0.12 (-2.82, 3.00) | PRV 40 |  |  |  |  |  |  |  |  |  |  |  |  |
| 0.92 (-1.30, 2.71) | -0.37 (-2.08, 1.37) | -0.69 (-2.70, 1.04) | -1.32 (-4.13, 1.80) | -1.81 (-3.67, 0.25) | -0.00 (-1.90, 2.83) | -0.62 (-3.07, 2.00) | -0.68 (-2.76, 1.61) | PTV 1 |  |  |  |  |  |  |  |  |  |  |  |
| 1.44 (-0.28, 2.90) | 0.18 (-0.96, 1.44) | -0.14 (-1.63, 1.14) | -0.76 (-3.55, 2.08) | -1.26 (-2.69, 0.39) | 0.54 (-0.97, 3.04) | -0.03 (-2.19, 2.31) | -0.11 (-1.86, 1.79) | 0.54 (-0.69, 1.80) | PTV 4 |  |  |  |  |  |  |  |  |  |  |
| 1.82 (-0.57, 3.38) | 0.64 (-1.61, 1.91) | 0.30 (-2.21, 1.62) | -0.29 (-3.56, 2.43) | -0.81 (-2.75, 0.06) | 0.16 (-0.88, 1.47) | 0.35 (-3.22, 3.11) | 0.29 (-1.95, 1.53) | 0.96 (-2.05, 2.83) | 0.44 (-2.32, 2.03) | RSV 10 |  |  |  |  |  |  |  |  |  |
| 2.19 (-0.53, 3.89) | 1.02 (-1.57, 2.39) | 0.36 (-2.46, 2.95) | 0.09 (-3.38, 2.69) | -0.41 (-2.73, 0.59) | 0.18 (-0.86, 1.55) | 0.70 (-3.07, 3.56) | 0.67 (-1.81, 2.00) | 1.35 (-1.97, 3.27) | 0.82 (-2.21, 2.43) | 0.42 (-0.77, 1.23) | RSV 20 |  |  |  |  |  |  |  |  |
| 2.05 (0.01, 3.43) | 0.79 (-0.98, 1.98) | 0.47 (-1.55, 1.72) | -0.06 (-3.14, 2.64) | -0.63 (-2.05, 0.18) | 1.13 (-0.22, 3.56) | 0.57 (-2.65, 3.35) | 0.49 (-1.31, 1.69) | 1.14 (-1.38, 2.98) | 0.61 (-1.63, 2.14) | 0.12 (-0.52, 1.55) | -0.25 (-1.13, 1.67) | RSV 40 |  |  |  |  |  |  |  |
| 3.01 (-0.60, 7.11) | 1.88 (-1.74, 5.48) | 1.53 (-2.16, 5.16) | 0.86 (-3.96, 5.65) | 0.46 (-3.16, 4.02) | 2.33 (-0.86, 5.66) | 1.51 (-2.81, 6.22) | 1.46 (-2.10, 5.42) | 2.21 (-1.76, 6.24) | 1.63 (-2.18, 5.44) | 1.41 (-2.32, 5.30) | 1.09 (-2.80, 5.10) | 1.17 (-2.48, 4.91) | SIV 20 |  |  |  |  |  |  |
| **Subgroup analysis of dyslipidemia** | | | | | | | | | | | | | | | | | | | |
| ATV 10 |  |  |  |  |  |  |  |  |  |  |  |  |  |  |  |  |  |  |  |
| -0.15 (-20.85, 20.24) | ATV 20 |  |  |  |  |  |  |  |  |  |  |  |  |  |  |  |  |  |  |
| 0.16 (-22.27, 22.62) | 0.43 (-16.14, 16.85) | ATV 40 |  |  |  |  |  |  |  |  |  |  |  |  |  |  |  |  |  |
| 1.98 (-10.76, 14.32) | 2.19 (-13.96, 18.42) | 1.75 (-17.23, 20.83) | ATV 80 |  |  |  |  |  |  |  |  |  |  |  |  |  |  |  |  |
| -2.85 (-20.90, 15.02) | -2.71 (-13.53, 8.12) | -3.05 (-17.28, 11.32) | -4.78 (-18.22, 8.52) | Control |  |  |  |  |  |  |  |  |  |  |  |  |  |  |  |
| -2.74 (-24.90, 18.99) | -2.68 (-19.18, 14.05) | -2.95 (-21.45, 15.89) | -4.71 (-23.08, 12.89) | 0.07 (-12.32, 12.12) | PRV 10 |  |  |  |  |  |  |  |  |  |  |  |  |  |  |
| 0.45 (-17.16, 18.17) | 0.72 (-19.49, 20.28) | 0.35 (-22.43, 22.78) | -1.48 (-13.75, 10.71) | 3.28 (-14.61, 21.38) | 3.29 (-18.42, 25.27) | PRV 40 |  |  |  |  |  |  |  |  |  |  |  |  |  |
| 0.29 (-11.57, 12.17) | 0.55 (-23.61, 24.46) | 0.14 (-25.36, 26.09) | -1.70 (-18.98, 15.97) | 3.18 (-18.78, 25.13) | 3.08 (-21.61, 28.66) | -0.17 (-21.41, 22.10) | PTV 2 |  |  |  |  |  |  |  |  |  |  |  |  |
| 0.31 (-18.35, 19.16) | 0.44 (-10.70, 11.67) | 0.11 (-12.29, 12.08) | -1.61 (-16.08, 12.43) | 3.15 (-4.17, 10.24) | 2.95 (-11.31, 17.41) | -0.13 (-19.04, 18.84) | -0.01 (-22.69, 22.06) | RSV 10 |  |  |  |  |  |  |  |  |  |  |  |
| -1.03 (-22.19, 19.43) | -0.93 (-15.83, 13.78) | -1.31 (-17.63, 15.27) | -3.07 (-20.10, 13.51) | 1.75 (-9.81, 13.07) | 1.72 (-14.61, 18.61) | -1.53 (-22.83, 19.40) | -1.33 (-26.10, 22.61) | -1.40 (-12.61, 9.68) | RSV 20 |  |  |  |  |  |  |  |  |  |  |
| -1.29 (-21.94, 20.02) | -1.06 (-15.92, 14.37) | -1.46 (-17.88, 14.84) | -3.22 (-20.02, 13.51) | 1.60 (-9.94, 12.92) | 1.57 (-15.37, 18.50) | -1.62 (-22.20, 19.27) | -1.63 (-26.47, 23.16) | -1.51 (-12.82, 9.58) | -0.08 (-12.82, 12.10) | RSV 5 |  |  |  |  |  |  |  |  |  |
| -0.34 (-20.37, 19.43) | -0.22 (-14.51, 14.31) | -0.60 (-17.48, 16.87) | -2.32 (-18.27, 13.35) | 2.51 (-7.90, 12.93) | 2.33 (-13.67, 18.48) | -0.85 (-20.84, 19.55) | -0.75 (-24.24, 22.70) | -0.70 (-12.47, 11.43) | 0.74 (-14.30, 15.77) | 0.84 (-14.36, 16.29) | SIV 10 |  |  |  |  |  |  |  |  |
| -1.37 (-19.86, 17.35) | -1.08 (-13.31, 11.16) | -1.55 (-17.29, 13.99) | -3.18 (-17.54, 10.93) | 1.60 (-5.25, 8.41) | 1.53 (-12.58, 15.59) | -1.87 (-20.26, 17.14) | -1.51 (-23.97, 20.66) | -1.54 (-10.80, 7.80) | -0.10 (-13.21, 12.63) | -0.01 (-12.92, 13.02) | -0.88 (-11.62, 9.97) | SIV 20 |  |  |  |  |  |  |  |
| 1.91 (-15.26, 19.00) | 2.03 (-8.45, 12.89) | 1.69 (-12.93, 16.06) | -0.03 (-12.24, 12.46) | 4.71 (-0.54, 10.16) | 4.65 (-8.51, 18.19) | 1.35 (-15.57, 18.98) | 1.55 (-19.69, 22.77) | 1.62 (-5.49, 8.92) | 2.99 (-8.72, 15.27) | 3.14 (-8.75, 14.91) | 2.31 (-8.12, 12.64) | 3.14 (-4.05, 10.63) | SIV 40 |  |  |  |  |  |  |
| -0.95 (-21.03, 19.07) | -0.78 (-15.19, 13.99) | -1.09 (-18.76, 16.63) | -2.91 (-19.18, 13.30) | 1.94 (-8.42, 12.50) | 1.86 (-14.50, 17.88) | -1.50 (-21.31, 19.02) | -1.42 (-24.79, 22.13) | -1.27 (-13.36, 11.34) | 0.13 (-14.84, 15.43) | 0.25 (-14.79, 15.69) | -0.56 (-12.84, 11.87) | 0.34 (-10.31, 11.20) | -2.80 (-13.54, 7.84) | SIV 80 |  |  |  |  |  |
| **Subgroup analysis of ACS** | | | | | | | | | | | | | | | | | | | |
| ATV 20 |  |  |  |  |  |  |  |  |  |  |  |  |  |  |  |  |  |  |  |
| 1.20 (-4.94, 9.48) | ATV 40 |  |  |  |  |  |  |  |  |  |  |  |  |  |  |  |  |  |  |
| 0.67 (-10.31, 11.58) | -0.45 (-14.32, 11.67) | ATV 5 |  |  |  |  |  |  |  |  |  |  |  |  |  |  |  |  |  |
| 2.59 (-3.86, 11.48) | 1.35 (-7.65, 11.46) | 1.81 (-10.05, 16.46) | ATV 80 |  |  |  |  |  |  |  |  |  |  |  |  |  |  |  |  |
| -0.17 (-10.98, 9.32) | -1.57 (-12.89, 6.88) | -0.88 (-16.58, 13.20) | -2.96 (-13.87, 4.81) | Control |  |  |  |  |  |  |  |  |  |  |  |  |  |  |  |
| -0.29 (-15.50, 15.39) | -1.35 (-19.18, 14.63) | -0.95 (-19.77, 18.58) | -2.68 (-21.19, 12.97) | -0.22 (-17.43, 19.01) | PTV 1 |  |  |  |  |  |  |  |  |  |  |  |  |  |  |
| 0.21 (-10.96, 11.70) | -0.80 (-15.40, 11.47) | -0.43 (-16.09, 15.53) | -2.11 (-17.25, 10.06) | 0.37 (-14.03, 16.13) | 0.52 (-10.46, 11.53) | PTV 4 |  |  |  |  |  |  |  |  |  |  |  |  |  |
| 0.41 (-14.39, 14.07) | -0.81 (-16.43, 11.81) | -0.27 (-19.22, 17.08) | -2.12 (-18.07, 10.02) | 0.56 (-9.80, 11.01) | 0.74 (-21.44, 20.84) | 0.20 (-19.46, 17.61) | RSV 10 |  |  |  |  |  |  |  |  |  |  |  |  |
| 0.64 (-14.22, 14.26) | -0.58 (-16.22, 11.89) | -0.03 (-18.55, 17.12) | -1.92 (-17.76, 9.91) | 0.75 (-9.64, 10.83) | 0.93 (-20.86, 20.57) | 0.39 (-18.86, 17.22) | 0.21 (-7.47, 8.27) | RSV 20 |  |  |  |  |  |  |  |  |  |  |  |
| **Subgroup analysis of non-ACS** | | | | | | | | | | | | | | | | | | | |
| ATV 10 |  |  |  |  |  |  |  |  |  |  |  |  |  |  |  |  |  |  |  |
| 1.11 (-3.21, 5.53) | ATV 20 |  |  |  |  |  |  |  |  |  |  |  |  |  |  |  |  |  |  |
| 1.07 (-3.46, 5.42) | -0.05 (-4.14, 3.95) | ATV 40 |  |  |  |  |  |  |  |  |  |  |  |  |  |  |  |  |  |
| 2.34 (-1.46, 6.20) | 1.21 (-3.10, 5.70) | 1.32 (-3.11, 5.72) | ATV 80 |  |  |  |  |  |  |  |  |  |  |  |  |  |  |  |  |
| -1.02 (-4.60, 2.57) | -2.12 (-6.02, 1.83) | -2.08 (-5.96, 2.04) | -3.39 (-6.90, 0.16) | Control |  |  |  |  |  |  |  |  |  |  |  |  |  |  |  |
| -1.01 (-8.45, 6.68) | -2.08 (-9.70, 5.91) | -2.07 (-9.66, 5.87) | -3.36 (-10.80, 4.23) | 0.07 (-6.55, 6.69) | PRV 10 |  |  |  |  |  |  |  |  |  |  |  |  |  |  |
| 0.62 (-4.46, 5.76) | -0.48 (-6.04, 4.98) | -0.43 (-5.84, 5.11) | -1.73 (-5.77, 2.25) | 1.62 (-2.85, 6.14) | 1.63 (-6.59, 9.57) | PRV 40 |  |  |  |  |  |  |  |  |  |  |  |  |  |
| 0.30 (-6.24, 7.15) | -0.86 (-8.66, 7.37) | -0.79 (-8.87, 7.38) | -2.07 (-9.54, 5.77) | 1.29 (-6.13, 8.95) | 1.26 (-8.83, 11.39) | -0.36 (-8.50, 8.15) | PTV 2 |  |  |  |  |  |  |  |  |  |  |  |  |
| 1.34 (-2.46, 5.24) | 0.24 (-3.75, 4.43) | 0.33 (-3.69, 4.36) | -1.01 (-4.91, 2.95) | 2.37 (-0.81, 5.71) | 2.33 (-5.18, 9.83) | 0.73 (-4.25, 5.81) | 1.07 (-6.71, 8.58) | RSV 10 |  |  |  |  |  |  |  |  |  |  |  |
| 0.50 (-5.14, 6.33) | -0.56 (-6.22, 5.29) | -0.49 (-5.91, 4.99) | -1.84 (-7.56, 4.11) | 1.51 (-3.61, 6.88) | 1.52 (-7.03, 9.84) | -0.11 (-6.56, 6.56) | 0.28 (-8.73, 9.21) | -0.82 (-5.90, 4.35) | RSV 20 |  |  |  |  |  |  |  |  |  |  |
| 1.66 (-4.02, 7.44) | 0.52 (-5.36, 6.46) | 0.60 (-5.32, 6.57) | -0.72 (-5.78, 4.38) | 2.66 (-2.75, 8.18) | 2.58 (-5.94, 11.30) | 1.04 (-5.31, 7.25) | 1.34 (-7.38, 10.16) | 0.28 (-4.84, 5.33) | 1.09 (-6.02, 7.96) | RSV 40 |  |  |  |  |  |  |  |  |  |
| 0.31 (-6.17, 6.84) | -0.80 (-7.35, 5.86) | -0.76 (-7.22, 5.76) | -2.07 (-8.57, 4.45) | 1.33 (-4.53, 7.27) | 1.29 (-7.56, 10.44) | -0.31 (-7.66, 6.94) | 0.07 (-9.36, 9.10) | -1.09 (-6.79, 4.83) | -0.22 (-6.41, 6.13) | -1.36 (-8.87, 6.20) | RSV 5 |  |  |  |  |  |  |  |  |
| 1.32 (-5.21, 8.07) | 0.25 (-6.37, 7.22) | 0.28 (-6.53, 7.15) | -1.00 (-7.54, 5.57) | 2.35 (-3.28, 8.17) | 2.28 (-6.43, 11.24) | 0.71 (-6.44, 7.90) | 1.10 (-8.41, 10.30) | 0.00 (-6.45, 6.48) | 0.82 (-6.86, 8.38) | -0.25 (-8.10, 7.43) | 1.08 (-7.13, 9.16) | SIV 10 |  |  |  |  |  |  |  |
| 0.66 (-4.04, 5.43) | -0.44 (-5.28, 4.63) | -0.39 (-5.34, 4.74) | -1.68 (-6.29, 2.92) | 1.67 (-1.53, 5.00) | 1.66 (-5.91, 9.14) | 0.08 (-5.34, 5.42) | 0.40 (-7.79, 8.53) | -0.68 (-5.14, 3.71) | 0.17 (-5.95, 6.11) | -0.97 (-7.21, 5.38) | 0.35 (-6.23, 7.04) | -0.68 (-6.55, 5.07) | SIV 20 |  |  |  |  |  |  |
| 3.33 (-0.63, 7.47) | 2.22 (-1.72, 6.36) | 2.30 (-1.83, 6.69) | 0.97 (-2.71, 4.79) | 4.34 (1.76, 7.10) | 4.30 (-2.71, 11.55) | 2.71 (-2.00, 7.70) | 3.04 (-4.67, 10.71) | 1.98 (-1.36, 5.41) | 2.78 (-2.61, 8.30) | 1.71 (-3.86, 7.29) | 3.03 (-3.17, 9.32) | 1.99 (-3.78, 7.84) | 2.65 (-0.95, 6.52) | SIV 40 |  |  |  |  |  |
| 0.78 (-5.66, 7.37) | -0.33 (-6.79, 6.46) | -0.26 (-6.91, 6.63) | -1.56 (-8.04, 4.84) | 1.82 (-3.82, 7.47) | 1.75 (-7.02, 10.46) | 0.21 (-7.04, 7.10) | 0.51 (-8.62, 9.67) | -0.54 (-6.87, 5.65) | 0.25 (-7.18, 7.87) | -0.83 (-8.64, 6.72) | 0.46 (-7.48, 8.45) | -0.58 (-7.38, 6.19) | 0.10 (-5.79, 5.83) | -2.50 (-8.35, 3.13) | SIV 80 |  |  |  |  |
| **Subgroup analysis of < 12-month duration** | | | | | | | | | | | | | | | | | | | |
| ATV 10 |  |  |  |  |  |  |  |  |  |  |  |  |  |  |  |  |  |  |  |
| 0.40 (-3.61, 4.29) | ATV 20 |  |  |  |  |  |  |  |  |  |  |  |  |  |  |  |  |  |  |
| 1.25 (-2.90, 5.37) | 0.84 (-2.42, 4.18) | ATV 40 |  |  |  |  |  |  |  |  |  |  |  |  |  |  |  |  |  |
| 2.72 (-1.20, 6.76) | 2.32 (-1.10, 5.92) | 1.50 (-2.57, 5.64) | ATV 80 |  |  |  |  |  |  |  |  |  |  |  |  |  |  |  |  |
| -0.91 (-4.68, 2.53) | -1.34 (-4.71, 2.00) | -2.17 (-5.67, 1.20) | -3.66 (-7.37, -0.19) | Control |  |  |  |  |  |  |  |  |  |  |  |  |  |  |  |
| -0.94 (-8.43, 6.54) | -1.31 (-8.55, 6.15) | -2.15 (-9.59, 5.27) | -3.62 (-11.16, 3.86) | 0.03 (-6.64, 6.75) | PRV 10 |  |  |  |  |  |  |  |  |  |  |  |  |  |  |
| 0.62 (-9.85, 11.05) | 0.20 (-9.38, 9.64) | -0.61 (-10.99, 9.47) | -2.07 (-12.48, 7.92) | 1.53 (-8.58, 11.63) | 1.47 (-10.65, 13.76) | PRV 20 |  |  |  |  |  |  |  |  |  |  |  |  |  |
| 0.23 (-6.43, 6.80) | -0.13 (-7.92, 7.60) | -0.95 (-8.91, 6.76) | -2.44 (-10.28, 5.22) | 1.21 (-6.33, 8.61) | 1.13 (-8.75, 11.13) | -0.28 (-12.90, 11.67) | PTV 2 |  |  |  |  |  |  |  |  |  |  |  |  |
| 0.63 (-7.24, 8.27) | 0.24 (-6.47, 6.94) | -0.57 (-8.19, 6.84) | -2.05 (-9.75, 5.38) | 1.59 (-5.94, 9.03) | 1.55 (-8.27, 11.58) | 0.03 (-6.87, 6.95) | 0.38 (-9.85, 10.56) | PTV 4 |  |  |  |  |  |  |  |  |  |  |  |
| 0.92 (-2.94, 4.78) | 0.51 (-3.10, 4.31) | -0.32 (-3.85, 3.27) | -1.82 (-5.92, 2.28) | 1.86 (-1.02, 4.80) | 1.86 (-5.51, 9.10) | 0.32 (-9.99, 10.89) | 0.65 (-6.81, 8.53) | 0.31 (-7.44, 8.12) | RSV 10 |  |  |  |  |  |  |  |  |  |  |
| 0.55 (-4.15, 5.22) | 0.16 (-4.28, 4.60) | -0.68 (-4.92, 3.58) | -2.19 (-6.94, 2.49) | 1.48 (-2.14, 5.22) | 1.44 (-6.25, 9.16) | -0.01 (-10.66, 10.55) | 0.30 (-7.52, 8.58) | -0.08 (-8.07, 7.90) | -0.37 (-3.79, 3.03) | RSV 20 |  |  |  |  |  |  |  |  |  |
| 0.17 (-6.42, 6.76) | -0.28 (-6.62, 6.24) | -1.09 (-7.52, 5.42) | -2.58 (-9.27, 3.95) | 1.09 (-4.50, 7.01) | 1.08 (-7.82, 9.82) | -0.51 (-11.92, 11.33) | -0.12 (-9.20, 9.36) | -0.47 (-9.82, 8.97) | -0.74 (-6.40, 5.06) | -0.38 (-6.29, 5.47) | RSV 5 |  |  |  |  |  |  |  |  |
| 1.51 (-5.07, 8.07) | 1.06 (-5.42, 7.66) | 0.28 (-6.51, 6.92) | -1.22 (-8.07, 5.33) | 2.44 (-3.26, 8.21) | 2.44 (-6.62, 11.25) | 0.95 (-10.59, 12.54) | 1.24 (-8.19, 10.50) | 0.84 (-8.33, 10.35) | 0.58 (-5.75, 6.93) | 0.92 (-5.84, 7.78) | 1.33 (-6.88, 9.46) | SIV 10 |  |  |  |  |  |  |  |
| 0.77 (-4.14, 5.42) | 0.37 (-4.15, 4.83) | -0.48 (-5.17, 4.13) | -1.92 (-6.87, 2.63) | 1.71 (-1.61, 4.92) | 1.72 (-5.81, 9.10) | 0.15 (-10.30, 10.74) | 0.52 (-7.51, 8.83) | 0.15 (-7.87, 8.07) | -0.16 (-4.41, 3.99) | 0.20 (-4.68, 4.92) | 0.64 (-6.13, 7.02) | -0.73 (-6.66, 5.06) | SIV 20 |  |  |  |  |  |  |
| 3.35 (-0.94, 7.69) | 2.94 (-0.97, 7.06) | 2.09 (-1.90, 6.25) | 0.66 (-3.70, 4.87) | 4.29 (1.55, 7.18) | 4.27 (-2.86, 11.60) | 2.78 (-7.47, 13.37) | 3.09 (-4.62, 11.19) | 2.74 (-5.05, 10.53) | 2.46 (-0.92, 5.93) | 2.80 (-1.45, 7.15) | 3.20 (-2.99, 9.48) | 1.88 (-3.84, 7.79) | 2.60 (-1.01, 6.37) | SIV 40 |  |  |  |  |  |
| 0.90 (-5.88, 7.21) | 0.43 (-6.02, 6.88) | -0.40 (-7.06, 6.05) | -1.77 (-8.69, 4.56) | 1.79 (-3.87, 7.43) | 1.73 (-6.93, 10.51) | 0.28 (-11.50, 11.88) | 0.63 (-8.92, 10.03) | 0.22 (-9.14, 9.59) | -0.06 (-6.36, 6.22) | 0.32 (-6.51, 6.97) | 0.67 (-7.38, 8.68) | -0.59 (-7.18, 5.99) | 0.08 (-5.64, 5.99) | -2.48 (-8.25, 3.18) | SIV 80 |  |  |  |  |
| **Subgroup analysis of ≥ 12-month duration** | | | | | | | | | | | | | | | | | | | |
| **Consistency modle** | | | | | | | **Inonsistency modle** | | | | | |  |  |  |  |  |  |  |
| ATV 80 |  |  |  |  |  |  | ATV 80 |  |  |  |  |  |  |  |  |  |  |  |  |
| -2.13 (-4.24, -0.13) | Control |  |  |  |  |  | -2.14 (-4.32, -0.14) | Control |  |  |  |  |  |  |  |  |  |  |  |
| -1.27 (-2.56, -0.11) | 0.87 (-0.75, 2.54) | PRV 40 |  |  |  |  | -1.27 (-2.53, -0.02) | 0.88 (-0.76, 2.55) | PRV 40 |  |  |  |  |  |  |  |  |  |  |
| -0.46 (-2.85, 1.90) | 1.66 (-1.41, 4.90) | 0.79 (-1.80, 3.55) | RSV 10 |  |  |  | -0.49 (-2.87, 1.92) | 1.66 (-1.42, 4.84) | 0.76 (-1.84, 3.50) | RSV 10 |  |  |  |  |  |  |  |  |  |
| -0.46 (-2.19, 1.16) | 1.68 (-1.01, 4.39) | 0.80 (-1.32, 2.95) | 0.02 (-1.65, 1.71) | RSV 40 |  |  | -0.46 (-2.17, 1.24) | 1.67 (-0.97, 4.40) | 0.80 (-1.28, 2.89) | 0.02 (-1.60, 1.63) | RSV 40 |  |  |  |  |  |  |  |  |
| -0.11 (-1.80, 1.57) | 2.05 (-0.59, 4.74) | 1.18 (-0.86, 3.34) | 0.38 (-2.49, 3.22) | 0.38 (-1.95, 2.74) | SIV 40 |  | -0.12 (-1.78, 1.62) | 2.01 (-0.58, 4.73) | 1.13 (-0.90, 3.31) | 0.35 (-2.51, 3.39) | 0.34 (-2.01, 2.85) | SIV 40 |  |  |  |  |  |  |  |

ATV 10: Atorvastatin 10 mg/d; ATV 20: Atorvastatin 20 mg/d; ATV 40: Atorvastatin 40 mg/d; ATV 5: Atorvastatin 5 mg/d; ATV 80: Atorvastatin 80 mg/d; PRV 10: Pravastatin 10 mg/d; PRV 20: Pravastatin 20 mg/d; PRV 40: Pravastatin 40 mg/d; PTV 1: Pitavastatin 1 mg/d; PTV 2: Pitavastatin 2 mg/d; PTV 4: Pitavastatin 4 mg/d; RSV 10: Rosuvastatin 10 mg/d; RSV 20: Rosuvastatin 20 mg/d; RSV 40: Rosuvastatin 40 mg/d; RSV 5: Rosuvastatin 5 mg/d; SIV 10: Simvastatin 10 mg/d; SIV 20: Simvastatin 20 mg/d; SIV 40: Simvastatin 40 mg/d; SIV 80: Simvastatin 80 mg/d.
