# Supplementary material for: The effect of various types and doses of statins on C-reactive protein levels in patients with dyslipidemia or coronary heart disease: A systematic review and network meta-analysis
Source: Front Cardiovasc Med. 2022 Jul 27;9:936817. doi: 10.3389/fcvm.2022.936817 (PMC9363636; doi:10.3389/fcvm.2022.936817)
Supplement: Supplementary file 5 [file Table_5.docx]

**Supplementary Table 5. Results of node-splitting models.**

| **Items** | **Comparison** | **Direct Effect** | **Indirect Effect** | **Overall** | **P-value** |
| --- | --- | --- | --- | --- | --- |
| **All included interventions** | ATV 10, ATV 20 | -1.60 (-7.73, 4.38) | -0.13 (-4.05, 3.93) | -0.35 (-3.84, 3.28) | 0.67 |
|  | ATV 10, ATV 40 | -1.92 (-8.12, 4.09) | -1.25 (-5.61, 3.07) | -1.22 (-4.92, 2.51) | 0.86 |
|  | ATV 10, ATV 80 | -2.59 (-6.88, 1.75) | -2.80 (-7.25, 1.45) | -2.49 (-5.74, 0.66) | 0.94 |
|  | ATV 10, Control | 0.20 (-4.07, 4.52) | 1.19 (-2.65, 5.16) | 0.84 (-2.25, 3.98) | 0.72 |
|  | ATV 10, RSV 10 | 0.43 (-5.40, 6.36) | -1.63 (-5.65, 2.40) | -0.96 (-4.23, 2.34) | 0.55 |
|  | ATV 20, ATV 40 | -0.24 (-3.70, 3.25) | -1.97 (-7.35, 3.36) | -0.85 (-3.76, 2.02) | 0.59 |
|  | ATV 20, ATV 5 | -0.79 (-7.14, 5.46) | 0.29 (-10.90, 11.60) | -0.54 (-5.88, 4.86) | 0.86 |
|  | ATV 20, ATV 80 | -3.34 (-7.40, 0.35) | -0.36 (-4.62, 4.11) | -2.13 (-5.13, 0.68) | 0.29 |
|  | ATV 20, Control | 1.50 (-2.93, 6.01) | 1.45 (-1.96, 4.81) | 1.21 (-1.72, 4.06) | 0.98 |
|  | ATV 20, PTV 4 | -0.21 (-6.14, 5.79) | -1.39 (-12.96, 9.93) | -0.43 (-5.53, 4.87) | 0.84 |
|  | ATV 20, RSV 10 | -1.14 (-7.08, 4.91) | -0.10 (-3.66, 3.33) | -0.60 (-3.74, 2.43) | 0.76 |
|  | ATV 20, SIV 40 | -0.09 (-6.04, 5.77) | -3.68 (-7.53, -0.22) | -2.89 (-6.25, 0.34) | 0.29 |
|  | ATV 40, ATV 80 | -1.36 (-7.55, 4.80) | -1.31 (-5.07, 2.41) | -1.30 (-4.65, 1.96) | 0.99 |
|  | ATV 40, Control | 3.72 (-1.11, 8.71) | 1.21 (-2.41, 4.72) | 2.05 (-1.06, 5.18) | 0.41 |
|  | ATV 40, RSV 10 | 0.05 (-6.05, 5.94) | 0.39 (-3.46, 4.29) | 0.24 (-2.94, 3.45) | 0.92 |
|  | ATV 40, RSV 20 | 0.07 (-8.35, 8.39) | 0.85 (-3.44, 5.25) | 0.63 (-3.20, 4.44) | 0.88 |
|  | ATV 40, SIV 40 | -17.72 (-45.15, 8.77) | -1.71 (-5.29, 1.73) | -2.03 (-5.57, 1.48) | 0.22 |
|  | ATV 5, PTV 1 | 0.14 (-7.35, 7.50) | 1.06 (-9.52, 11.76) | 0.44 (-5.41, 6.43) | 0.89 |
|  | ATV 80, Control | 2.18 (-2.07, 6.28) | 4.11 (1.02, 7.37) | 3.32 (0.83, 6.02) | 0.44 |
|  | ATV 80, PRV 40 | 1.33 (-3.02, 5.70) | 2.70 (-3.79, 9.43) | 1.73 (-1.69, 5.22) | 0.72 |
|  | ATV 80, RSV 40 | 0.54 (-5.43, 6.61) | 1.77 (-4.93, 8.56) | 0.96 (-3.34, 5.43) | 0.77 |
|  | ATV 80, SIV 40 | 0.16 (-5.94, 6.20) | -0.93 (-4.48, 2.61) | -0.77 (-3.71, 2.33) | 0.75 |
|  | Control, PRV 40 | -0.84 (-6.91, 5.18) | -2.22 (-7.43, 2.74) | -1.62 (-5.41, 2.12) | 0.72 |
|  | Control, RSV 10 | -0.85 (-4.46, 2.54) | -2.05 (-5.46, 1.32) | -1.82 (-4.35, 0.63) | 0.61 |
|  | Control, RSV 20 | -0.09 (-4.32, 4.04) | -2.36 (-7.75, 2.78) | -1.42 (-4.64, 1.93) | 0.51 |
|  | Control, SIV 40 | -5.04 (-7.81, -2.33) | -2.16 (-6.89, 2.23) | -4.07 (-6.52, -1.77) | 0.28 |
|  | PTV 1, PTV 4 | -0.44 (-6.32, 5.38) | 0.72 (-10.69, 11.89) | -0.34 (-5.61, 4.78) | 0.86 |
|  | RSV 10, RSV 20 | -0.19 (-3.64, 3.17) | -0.29 (-9.22, 8.55) | 0.39 (-2.63, 3.52) | 0.99 |
|  | RSV 10, RSV 40 | 0.01 (-6.08, 5.91) | -1.34 (-8.27, 5.60) | -0.57 (-4.92, 3.80) | 0.75 |
|  | RSV 10, SIV 40 | 0.54 (-3.54, 4.58) | -4.25 (-7.96, -0.70) | -2.27 (-5.11, 0.53) | 0.08 |
|  | SIV 20, SIV 40 | -0.47 (-4.33, 3.31) | -5.18 (-9.26, -1.29) | -2.45 (-5.75, 0.67) | 0.09 |
| **Subgroup analysis of CRP** | No closed loop. |  |  |  |  |
| **Subgroup analysis of hs-CRP** | ATV 10, ATV 20 | -1.70 (-8.27, 5.18) | -0.12 (-4.45, 4.38) | -0.36 (-4.36, 3.59) | 0.7 |
|  | ATV 10, ATV 40 | -1.85 (-8.64, 4.69) | -1.30 (-6.00, 3.47) | -1.20 (-5.29, 2.82) | 0.89 |
|  | ATV 10, ATV 80 | -2.65 (-7.49, 2.33) | -3.40 (-8.72, 1.61) | -2.79 (-6.61, 0.90) | 0.81 |
|  | ATV 10, Control | 0.24 (-4.61, 4.92) | 1.37 (-3.21, 5.95) | 0.89 (-2.60, 4.37) | 0.73 |
|  | ATV 10, RSV 10 | 0.42 (-6.23, 7.12) | -1.35 (-6.00, 3.31) | -0.82 (-4.58, 2.92) | 0.65 |
|  | ATV 20, ATV 40 | -0.22 (-4.11, 3.70) | -1.81 (-7.84, 4.12) | -0.82 (-4.04, 2.26) | 0.65 |
|  | ATV 20, ATV 5 | -0.87 (-7.83, 6.26) | 0.33 (-11.80, 12.49) | -0.53 (-6.42, 5.28) | 0.86 |
|  | ATV 20, ATV 80 | -3.46 (-7.79, 0.58) | -0.54 (-5.70, 4.71) | -2.43 (-5.74, 0.77) | 0.36 |
|  | ATV 20, Control | 1.68 (-3.26, 6.41) | 1.63 (-2.18, 5.40) | 1.23 (-1.94, 4.47) | 0.99 |
|  | ATV 20, PTV 4 | -0.20 (-6.88, 6.38) | -1.26 (-14.02, 11.22) | -0.49 (-6.25, 5.15) | 0.89 |
|  | ATV 20, RSV 10 | -1.22 (-7.80, 5.49) | 0.01 (-4.10, 3.94) | -0.44 (-3.93, 3.15) | 0.74 |
|  | ATV 20, SIV 40 | -0.10 (-6.76, 6.56) | -3.81 (-7.96, 0.13) | -2.85 (-6.65, 0.67) | 0.31 |
|  | ATV 40, ATV 80 | -1.28 (-8.11, 5.48) | -1.73 (-6.27, 2.68) | -1.61 (-5.38, 2.10) | 0.91 |
|  | ATV 40, Control | 3.75 (-1.50, 9.22) | 1.33 (-2.75, 5.27) | 2.10 (-1.20, 5.46) | 0.46 |
|  | ATV 40, RSV 10 | -0.02 (-6.63, 6.68) | 0.61 (-3.61, 4.84) | 0.39 (-3.22, 3.78) | 0.87 |
|  | ATV 40, RSV 20 | -0.04 (-8.90, 8.78) | 0.83 (-4.01, 5.83) | 0.78 (-3.41, 5.07) | 0.87 |
|  | ATV 40, SIV 40 | -16.28 (-43.63, 11.48) | -1.67 (-5.57, 2.21) | -2.03 (-5.92, 1.64) | 0.32 |
|  | ATV 5, PTV 1 | 0.04 (-8.05, 7.91) | 1.06 (-10.83, 13.18) | 0.37 (-6.11, 6.86) | 0.87 |
|  | ATV 80, Control | 2.09 (-2.59, 6.88) | 4.86 (0.96, 9.08) | 3.66 (0.58, 7.01) | 0.36 |
|  | ATV 80, SIV 40 | 0.04 (-6.47, 6.79) | -0.69 (-5.04, 3.65) | -0.43 (-3.99, 3.07) | 0.84 |
|  | Control, RSV 10 | -0.55 (-4.42, 3.30) | -1.85 (-5.89, 2.19) | -1.68 (-4.58, 1.17) | 0.63 |
|  | Control, RSV 20 | -0.13 (-4.82, 4.68) | -2.29 (-8.12, 3.59) | -1.30 (-4.98, 2.27) | 0.54 |
|  | Control, SIV 40 | -5.12 (-8.28, -2.14) | -2.50 (-7.85, 2.40) | -4.10 (-6.83, -1.60) | 0.36 |
|  | PTV 1, PTV 4 | -0.53 (-7.10, 6.17) | 0.56 (-12.09, 13.15) | -0.32 (-6.08, 5.49) | 0.88 |
|  | RSV 10, RSV 20 | -0.18 (-3.97, 3.66) | -0.28 (-9.61, 9.21) | 0.38 (-2.98, 3.77) | 0.98 |
|  | RSV 10, SIV 40 | 0.45 (-3.90, 4.99) | -4.57 (-8.79, -0.58) | -2.40 (-5.73, 0.82) | 0.09 |
|  | SIV 20, SIV 40 | -0.47 (-4.75, 3.74) | -5.96 (-10.62, -1.50) | -2.76 (-6.79, 1.03) | 0.08 |
| **Subgroup analysis of CRP/hs-CRP with clear measurement method** | ATV 10, ATV 80 | -2.04 (-9.87, 5.79) | -1.07 (-8.51, 6.51) | -1.61 (-6.72, 3.58) | 0.86 |
|  | ATV 10, Control | 0.25 (-7.68, 8.15) | 1.81 (-4.63, 8.24) | 1.18 (-3.34, 5.82) | 0.75 |
|  | ATV 10, RSV 10 | 0.34 (-7.37, 8.15) | -1.95 (-9.02, 5.17) | -0.94 (-5.76, 3.95) | 0.63 |
|  | ATV 20, ATV 40 | -0.41 (-8.07, 7.48) | -0.58 (-8.99, 7.80) | -0.48 (-5.96, 4.96) | 0.97 |
|  | ATV 20, Control | 1.36 (-6.63, 9.12) | 2.72 (-3.30, 8.90) | 2.31 (-2.92, 7.93) | 0.76 |
|  | ATV 20, RSV 10 | -1.13 (-9.12, 6.64) | 0.90 (-5.66, 7.33) | 0.20 (-5.12, 5.64) | 0.67 |
|  | ATV 20, SIV 40 | -0.03 (-7.59, 7.51) | -3.46 (-9.81, 2.95) | -1.95 (-7.54, 3.65) | 0.46 |
|  | ATV 40, Control | 7.74 (-2.14, 17.54) | 1.31 (-4.38, 6.74) | 2.81 (-1.88, 7.71) | 0.25 |
|  | ATV 40, RSV 10 | -0.05 (-7.86, 7.84) | 1.11 (-5.14, 7.61) | 0.71 (-3.92, 5.38) | 0.81 |
|  | ATV 40, RSV 20 | 0.04 (-9.21, 9.72) | 2.03 (-4.61, 8.85) | 1.40 (-3.95, 6.88) | 0.73 |
|  | ATV 40, SIV 40 | -18.90 (-42.62, 7.84) | -1.04 (-6.38, 4.57) | -1.49 (-6.69, 3.78) | 0.18 |
|  | ATV 80, Control | 1.00 (-6.76, 8.60) | 3.70 (-1.77, 9.17) | 2.78 (-1.50, 7.06) | 0.55 |
|  | ATV 80, PRV 40 | 1.47 (-6.42, 9.60) | 1.96 (-7.27, 11.23) | 1.69 (-3.88, 7.34) | 0.94 |
|  | ATV 80, SIV 40 | 0.13 (-7.55, 7.95) | -2.46 (-8.69, 3.66) | -1.49 (-6.09, 2.95) | 0.58 |
|  | Control, PRV 40 | -0.88 (-8.83, 6.92) | -1.39 (-10.49, 7.78) | -1.07 (-6.65, 4.54) | 0.93 |
|  | Control, RSV 10 | -0.96 (-5.35, 3.59) | -3.03 (-8.45, 2.17) | -2.09 (-5.54, 1.03) | 0.51 |
|  | Control, RSV 20 | -0.25 (-5.56, 5.04) | -3.19 (-14.60, 7.77) | -1.41 (-5.82, 3.02) | 0.62 |
|  | Control, SIV 40 | -5.19 (-8.83, -1.82) | -2.19 (-8.54, 3.77) | -4.28 (-7.21, -1.43) | 0.37 |
|  | RSV 10, RSV 20 | -0.06 (-5.35, 5.33) | -0.95 (-12.08, 10.05) | 0.72 (-3.76, 5.18) | 0.89 |
|  | RSV 10, SIV 40 | 0.48 (-4.75, 5.69) | -4.44 (-9.41, 0.27) | -2.16 (-5.89, 1.55) | 0.15 |
|  | SIV 20, SIV 40 | -0.49 (-5.52, 4.34) | -5.50 (-10.66, -0.68) | -2.64 (-6.63, 1.33) | 0.14 |
| **Subgroup analysis of CHD** | ATV 10, ATV 20 | -1.57 (-4.07, 0.74) | -0.62 (-2.47, 1.61) | -0.98 (-2.47, 0.57) | 0.46 |
|  | ATV 10, ATV 40 | -1.81 (-4.41, 0.73) | -1.46 (-3.96, 0.93) | -1.47 (-3.12, 0.09) | 0.83 |
|  | ATV 10, ATV 80 | -2.73 (-4.04, -1.24) | -1.78 (-3.47, -0.46) | -2.25 (-3.65, -0.97) | 0.31 |
|  | ATV 10, Control | 0.26 (-0.43, 0.92) | -1.76 (-2.70, -0.51) | -0.16 (-1.43, 1.19) | 0.01^*^ |
|  | ATV 20, ATV 40 | -0.24 (-1.49, 0.99) | -3.28 (-8.08, 1.44) | -0.48 (-1.81, 0.64) | 0.22 |
|  | ATV 20, ATV 80 | -1.65 (-2.68, -0.85) | 0.56 (-0.78, 1.91) | -1.27 (-2.65, -0.08) | 0.01^*^ |
|  | ATV 20, Control | 1.74 (-0.33, 3.90) | 0.32 (-1.31, 1.94) | 0.81 (-0.59, 2.22) | 0.23 |
|  | ATV 40, ATV 80 | -1.32 (-3.08, 0.51) | -0.34 (-1.88, 1.08) | -0.78 (-2.19, 0.61) | 0.33 |
|  | ATV 40, Control | 2.37 (0.64, 4.71) | 0.44 (-1.47, 2.07) | 1.31 (-0.08, 2.90) | 0.09 |
|  | ATV 40, RSV 20 | -0.10 (-6.53, 6.39) | 0.88 (-1.49, 3.47) | 0.77 (-1.13, 3.72) | 0.78 |
|  | ATV 80, Control | 2.03 (0.35, 3.82) | 2.31 (0.63, 4.44) | 2.08 (1.08, 3.33) | 0.78 |
|  | ATV 80, PRV 40 | 1.10 (-1.26, 3.37) | 1.22 (-1.25, 4.13) | 1.16 (-0.33, 2.75) | 0.93 |
|  | ATV 80, RSV 40 | 0.46 (-1.41, 2.33) | 2.95 (0.03, 6.05) | 1.09 (-0.51, 3.04) | 0.14 |
|  | Control, PRV 40 | -0.88 (-3.21, 1.56) | -1.04 (-3.93, 1.58) | -0.93 (-2.49, 0.57) | 0.92 |
|  | Control, RSV 10 | 0.06 (-2.37, 3.12) | -0.61 (-3.15, 1.95) | -0.44 (-2.00, 2.00) | 0.62 |
|  | Control, RSV 20 | -0.15 (-2.19, 1.94) | -1.18 (-3.65, 1.24) | -0.58 (-2.22, 2.03) | 0.45 |
|  | RSV 10, RSV 20 | -0.24 (-1.75, 1.12) | -0.94 (-7.45, 5.67) | -0.12 (-1.47, 1.30) | 0.84 |
|  | RSV 10, RSV 40 | -0.02 (-1.87, 1.78) | -2.18 (-5.09, 0.72) | -0.60 (-2.64, 0.92) | 0.18 |
| **Subgroup analysis of** **dyslipidemia** | Control, RSV 10 | -1.24 (-10.96, 7.99) | -5.41 (-20.20, 9.05) | -3.15 (-10.24, 4.17) | 0.59 |
|  | Control, SIV 40 | -5.46 (-11.54, 0.39) | -1.49 (-17.06, 14.04) | -4.71 (-10.16, 0.54) | 0.6 |
|  | RSV 10, SIV 40 | 0.47 (-8.48, 9.22) | -4.83 (-15.55, 6.00) | -1.62 (-8.92, 5.49) | 0.4 |
|  | SIV 20, SIV 40 | -0.55 (-8.37, 7.19) | -7.56 (-16.27, 1.38) | -3.14 (-10.63, 4.05) | 0.21 |
| **Subgroup analysis of** **ACS** | ATV 20, ATV 40 | -0.28 (-7.67, 6.67) | -10.25 (-30.26, 4.55) | -1.20 (-9.48, 4.94) | 0.14 |
|  | ATV 20, ATV 80 | -3.12 (-13.23, 1.38) | 6.44 (-9.89, 22.11) | -2.59 (-11.48, 3.86) | 0.12 |
|  | ATV 40, Control | 7.70 (-3.09, 18.60) | -1.51 (-18.40, 8.71) | 1.57 (-6.88, 12.89) | 0.12 |
|  | ATV 80, Control | 0.98 (-8.90, 10.15) | 10.83 (-1.76, 27.36) | 2.96 (-4.81, 13.87) | 0.13 |
| **Subgroup analysis of** **non-ACS** | ATV 10, ATV 20 | -1.64 (-8.43, 5.14) | -1.03 (-6.58, 4.59) | -1.11 (-5.53, 3.21) | 0.89 |
|  | ATV 10, ATV 40 | -1.91 (-8.92, 5.06) | -0.66 (-6.23, 5.10) | -1.07 (-5.42, 3.46) | 0.77 |
|  | ATV 10, ATV 80 | -2.58 (-7.49, 2.20) | -2.39 (-8.56, 3.47) | -2.34 (-6.20, 1.46) | 0.96 |
|  | ATV 10, Control | 0.22 (-4.71, 4.97) | 1.64 (-3.35, 6.62) | 1.02 (-2.57, 4.60) | 0.66 |
|  | ATV 10, RSV 10 | 0.38 (-6.30, 7.07) | -2.31 (-6.94, 2.62) | -1.34 (-5.24, 2.46) | 0.51 |
|  | ATV 20, ATV 40 | -0.24 (-5.05, 4.54) | 0.64 (-6.40, 8.22) | 0.05 (-3.95, 4.14) | 0.83 |
|  | ATV 20, ATV 80 | -1.48 (-8.13, 5.34) | -1.12 (-6.52, 4.32) | -1.21 (-5.70, 3.10) | 0.92 |
|  | ATV 20, Control | 1.57 (-3.29, 6.52) | 2.72 (-2.01, 7.35) | 2.12 (-1.83, 6.02) | 0.74 |
|  | ATV 20, RSV 10 | -1.23 (-8.07, 5.71) | 0.33 (-4.33, 5.01) | -0.24 (-4.43, 3.75) | 0.71 |
|  | ATV 20, SIV 40 | -0.05 (-6.74, 6.70) | -3.19 (-7.76, 1.28) | -2.22 (-6.36, 1.72) | 0.42 |
|  | ATV 40, ATV 80 | -1.34 (-8.12, 5.68) | -1.08 (-6.42, 4.25) | -1.32 (-5.72, 3.11) | 0.95 |
|  | ATV 40, Control | 1.97 (-4.84, 9.03) | 2.00 (-2.60, 6.46) | 2.08 (-2.04, 5.96) | 1 |
|  | ATV 40, RSV 10 | -0.04 (-6.76, 6.65) | -0.55 (-5.66, 4.37) | -0.33 (-4.36, 3.69) | 0.9 |
|  | ATV 40, RSV 20 | 0.22 (-8.80, 9.18) | 0.72 (-6.38, 7.85) | 0.49 (-4.99, 5.91) | 0.92 |
|  | ATV 40, SIV 40 | -21.20 (-49.53, 15.49) | -1.91 (-6.27, 2.37) | -2.30 (-6.69, 1.83) | 0.28 |
|  | ATV 80, Control | 3.32 (-3.52, 10.09) | 3.50 (-0.38, 7.41) | 3.39 (-0.16, 6.90) | 0.95 |
|  | ATV 80, PRV 40 | 1.25 (-3.57, 6.08) | 2.83 (-5.09, 10.53) | 1.73 (-2.25, 5.77) | 0.72 |
|  | ATV 80, RSV 40 | 0.45 (-6.48, 7.21) | 1.16 (-6.96, 9.25) | 0.72 (-4.38, 5.78) | 0.9 |
|  | ATV 80, SIV 40 | 0.14 (-6.74, 6.77) | -1.41 (-6.37, 3.18) | -0.97 (-4.79, 2.71) | 0.7 |
|  | Control, PRV 40 | -0.83 (-7.73, 5.89) | -2.40 (-8.73, 3.78) | -1.62 (-6.14, 2.85) | 0.73 |
|  | Control, RSV 10 | -1.27 (-6.10, 3.47) | -2.63 (-6.95, 1.82) | -2.37 (-5.71, 0.81) | 0.67 |
|  | Control, RSV 20 | -0.31 (-6.97, 6.46) | -2.13 (-11.95, 7.85) | -1.51 (-6.88, 3.61) | 0.76 |
|  | Control, SIV 40 | -5.12 (-8.30, -2.13) | -2.32 (-7.87, 2.94) | -4.34 (-7.10, -1.76) | 0.36 |
|  | RSV 10, RSV 20 | -0.03 (-7.01, 6.77) | 0.28 (-9.62, 10.47) | 0.82 (-4.35, 5.90) | 0.94 |
|  | RSV 10, RSV 40 | 0.02 (-6.88, 6.67) | -0.60 (-8.94, 7.55) | -0.28 (-5.33, 4.84) | 0.91 |
|  | RSV 10, SIV 40 | 0.55 (-4.07, 5.15) | -4.06 (-8.60, 0.35) | -1.98 (-5.41, 1.36) | 0.14 |
|  | SIV 20, SIV 40 | -0.51 (-4.75, 3.75) | -5.67 (-10.14, -1.38) | -2.65 (-6.52, 0.95) | 0.09 |
| **Subgroup analysis of < 12-month duration** | ATV 10, ATV 20 | -1.70 (-8.71, 5.17) | -0.06 (-4.61, 4.56) | -0.40 (-4.29, 3.61) | 0.69 |
|  | ATV 10, ATV 40 | -1.90 (-8.84, 4.93) | -1.22 (-6.11, 3.63) | -1.25 (-5.37, 2.90) | 0.86 |
|  | ATV 10, ATV 80 | -2.55 (-7.49, 2.33) | -3.41 (-9.48, 2.41) | -2.72 (-6.76, 1.20) | 0.83 |
|  | ATV 10, Control | 0.22 (-4.83, 5.15) | 1.34 (-3.53, 6.25) | 0.91 (-2.53, 4.68) | 0.74 |
|  | ATV 10, RSV 10 | 0.38 (-6.44, 7.26) | -1.60 (-6.53, 3.39) | -0.92 (-4.78, 2.94) | 0.62 |
|  | ATV 20, ATV 40 | -0.28 (-4.22, 3.81) | -1.95 (-8.32, 4.06) | -0.84 (-4.18, 2.42) | 0.64 |
|  | ATV 20, ATV 80 | -3.45 (-7.99, 0.72) | 0.47 (-5.81, 6.49) | -2.32 (-5.92, 1.10) | 0.29 |
|  | ATV 20, Control | 1.60 (-3.36, 6.71) | 1.60 (-2.40, 5.53) | 1.34 (-2.00, 4.71) | 1.00 |
|  | ATV 20, RSV 10 | -1.09 (-7.96, 5.72) | 0.08 (-4.12, 4.23) | -0.51 (-4.31, 3.10) | 0.76 |
|  | ATV 20, SIV 40 | -0.08 (-6.98, 6.73) | -4.22 (-8.82, 0.13) | -2.94 (-7.06, 0.97) | 0.29 |
|  | ATV 40, ATV 80 | -1.35 (-8.35, 5.68) | -1.55 (-6.56, 3.12) | -1.50 (-5.64, 2.57) | 0.96 |
|  | ATV 40, Control | 3.85 (-1.54, 9.51) | 1.25 (-2.99, 5.45) | 2.17 (-1.20, 5.67) | 0.44 |
|  | ATV 40, RSV 10 | -0.06 (-7.14, 6.91) | 0.44 (-3.99, 4.74) | 0.32 (-3.27, 3.85) | 0.90 |
|  | ATV 40, RSV 20 | 0.15 (-9.02, 9.29) | 0.87 (-4.16, 5.90) | 0.68 (-3.58, 4.92) | 0.90 |
|  | ATV 40, SIV 40 | -16.49 (-45.25, 10.62) | -1.88 (-6.09, 2.30) | -2.09 (-6.25, 1.90) | 0.31 |
|  | ATV 80, Control | 2.11 (-2.73, 7.05) | 5.09 (0.39, 10.00) | 3.66 (0.19, 7.37) | 0.38 |
|  | Control, RSV 10 | -0.95 (-4.98, 3.09) | -2.01 (-6.17, 2.08) | -1.86 (-4.80, 1.02) | 0.70 |
|  | Control, RSV 20 | 0.05 (-4.87, 5.05) | -2.27 (-8.35, 4.02) | -1.48 (-5.22, 2.14) | 0.53 |
|  | Control, SIV 40 | -5.13 (-8.46, -2.01) | -1.79 (-9.24, 5.06) | -4.29 (-7.18, -1.55) | 0.39 |
|  | RSV 10, RSV 20 | -0.24 (-4.23, 3.71) | -0.33 (-10.29, 9.65) | 0.37 (-3.03, 3.79) | 0.99 |
|  | RSV 10, SIV 40 | 0.50 (-4.03, 5.04) | -5.23 (-9.78, -0.90) | -2.46 (-5.93, 0.92) | 0.07 |
|  | SIV 20, SIV 40 | -0.50 (-4.93, 3.90) | -5.94 (-10.58, -1.40) | -2.60 (-6.37, 1.01) | 0.09 |
| **Subgroup analysis of ≥ 12-month duration** | No closed loop. |  |  |  |  |

^*^ *P*<0.05. ATV 10: Atorvastatin 10 mg/d; ATV 20: Atorvastatin 20 mg/d; ATV 40: Atorvastatin 40 mg/d; ATV 5: Atorvastatin 5 mg/d; ATV 80: Atorvastatin 80 mg/d; PRV 10: Pravastatin 10 mg/d; PRV 20: Pravastatin 20 mg/d; PRV 40: Pravastatin 40 mg/d; PTV 1: Pitavastatin 1 mg/d; PTV 2: Pitavastatin 2 mg/d; PTV 4: Pitavastatin 4 mg/d; RSV 10: Rosuvastatin 10 mg/d; RSV 20: Rosuvastatin 20 mg/d; RSV 40: Rosuvastatin 40 mg/d; RSV 5: Rosuvastatin 5 mg/d; SIV 10: Simvastatin 10 mg/d; SIV 20: Simvastatin 20 mg/d; SIV 40: Simvastatin 40 mg/d; SIV 80: Simvastatin 80 mg/d.
