# Supplementary material for: The effect of various types and doses of statins on C-reactive protein levels in patients with dyslipidemia or coronary heart disease: A systematic review and network meta-analysis
Source: Front Cardiovasc Med. 2022 Jul 27;9:936817. doi: 10.3389/fcvm.2022.936817 (PMC9363636; doi:10.3389/fcvm.2022.936817)
Supplement: Supplementary file 6 [file Data_Sheet_1.docx]

**Supplementary Text 1. Search strategies.**

**1. PubMed：**

**#1** (((((((((((hyperlipemia) OR (hyperlipaemia)) OR (hyperlipemia)) OR (hyperlipidaemia)) OR (hyperlipidaemias)) OR (hyperlipidemia)) OR (hyperlipidemias)) OR (hyperlipidemic)) OR (lipaemia)) OR (lipemia)) OR (lipidaemia)) OR (lipidemia)

**#2** ((((((dyslipidemia) OR (dyslipaemia)) OR (dyslipemia)) OR (dyslipidaemia)) OR (dyslipidaemias)) OR (dyslipidemia)) OR (dyslipidemias)

**#3** ((((((hypertriglyceridemia) OR (hypertriglyceridaemia)) OR (hypertriglyceridemia)) OR (idiopathic familiary hypertriglyceridaemia)) OR (idiopathic familiary? hypertriglyceridemia)) OR (triglyceride storage disease)) OR (triglyceridemia)

**#4** ((((((((hypercholesteremia) OR (cholesteremia)) OR (cholesterinemia)) OR (cholesterolemia)) OR (hypercholesteremia)) OR (hypercholesterinaemia)) OR (hypercholesterinemia)) OR (hypercholesterolaemia)) OR (hypercholesterolemia)

**#5** ((((((((atherosclerosis) OR (atherosclerotic)) OR (atheromatous)) OR (Coronary heart disease)) OR (Coronary artery disease)) OR (ischemic heart disease)) OR (Acute coronary syndrome)) OR (Stable Angina)) OR (unstable angina)

**#6** #1 OR #2 OR #3 OR #4 OR #5

**#7** (((((((((((Statin) OR (Statins)) OR (Hydroxymethylglutaryl-CoA Reductase Inhibitors)) OR (3-Hydroxy-3-methylglutaryl coenzyme A reductase inhibitors)) OR (HMG-CoA reductase inhibitors)) OR (Atorvastatin)) OR (Fluvastatin)) OR (Lovastatin)) OR (Pravastatin)) OR (Rosuvastatin)) OR (Simvastatin)) OR (Pitavastatin)

**#8** ((C reactive protein) OR (CRP)) OR (hs-crp)

**#9** (randomized controlled trial[pt] OR controlled clinical trial[pt] OR randomized[tiab] OR placebo[tiab] OR clinical trials as topic[mesh:noexp] OR randomly[tiab] OR trial[ti]) NOT (animals [mh] NOT (humans [mh] AND animals[mh]))

**#10** #6 AND #7 AND #8 AND #9

**2. Cochrane library：**

#1 Atherosclerosis [MeSH]

#2 Hyperlipidemia [MeSH]

#3 Dyslipidemias [MeSH]

#4 Hypertriglyceridemia [MeSH]

#5 Hypercholesterolemia [MeSH]

#6 Coronary heart disease [MeSH]

#7 Coronary heart disease

#8 Myocardial Ischemia [MeSH]

#9 Acute coronary syndrome [MeSH]

#10 Angina, Stable [MeSH]

#11 Angina, Unstable [MeSH]

#12 #1 OR #2 OR #3 OR #4 OR #5 OR #6 OR #7 OR #8 OR #9 OR #10 OR #11

#13 Hydroxymethylglutaryl-CoA Reductase Inhibitors [MeSH]

#14 Atorvastatin [MeSH]

#15 Fluvastatin [MeSH]

#16 Lovastatin [MeSH]

#17 Pravastatin [MeSH]

#18 Rosuvastatin Calcium [MeSH]

#19 Simvastatin [MeSH]

#20 Pitavastatin

#21 #13 OR #14 OR #15 OR #16 OR #17 OR #18 OR #19 OR #20

#22 C-Reactive Protein [MeSH]

#23 CRP

#24 hs CRP

#25 #22 OR #23 OR #24

#26 #12 AND #21 AND #25

**3. Embase: (PICOs search strategy):**

**Population:** hyperlipidemia /exp OR dyslipidemia /exp OR hypertriglyceridemia /exp OR hypercholesterolemia /exp OR atherosclerosis /exp OR ischemic heart disease /exp OR coronary artery disease /exp OR acute coronary syndrome /exp OR stable angina pectoris /exp OR unstable angina pectoris /exp

**Intervention:** hydroxymethylglutaryl coenzyme A reductase Inhibitor /exp OR statin (proteln) /exp OR 3 hydroxy 3 methylglutaryl coenzyme a reductase Inhibrtor /exp OR atorvastatin /exp OR flulndostatin /exp OR mevinolln /exp OR pravastatin /exp OR rosuvastatin /exp OR simvastatin /exp OR pitavastatin /exp

**Outcome:** C reactive protein /exp

**Study design:** randomized controlled trial /exp

**4. OPENGREY:**

Atorvastatin or Fluvastatin or Lovastatin or Pravastatin or Rosuvastatin or Simvastatin or Pitavastatin or statin or statins

**5. ClinicalTrials.gov:**

**Intervention/treatment:**Atorvastatin OR Fluvastatin OR Lovastatin OR Pravastatin OR Rosuvastatin OR Simvastatin OR Pitavastatin OR statin OR statins

**Outcome Measure:** C reactive protein OR CRP OR hs CRP OR hs-CRP OR high-sensitive CRP

**Study Type:** Interventional (Clinical Trial)

**Study Results:** With results.
