# Supplementary material for: The effect of various types and doses of statins on C-reactive protein levels in patients with dyslipidemia or coronary heart disease: A systematic review and network meta-analysis
Source: Front Cardiovasc Med. 2022 Jul 27;9:936817. doi: 10.3389/fcvm.2022.936817 (PMC9363636; doi:10.3389/fcvm.2022.936817)
Supplement: Supplementary file 7 [file Image_1.pdf]

Supplementary Figure 1. Results of ranks for various types and doses of statins.

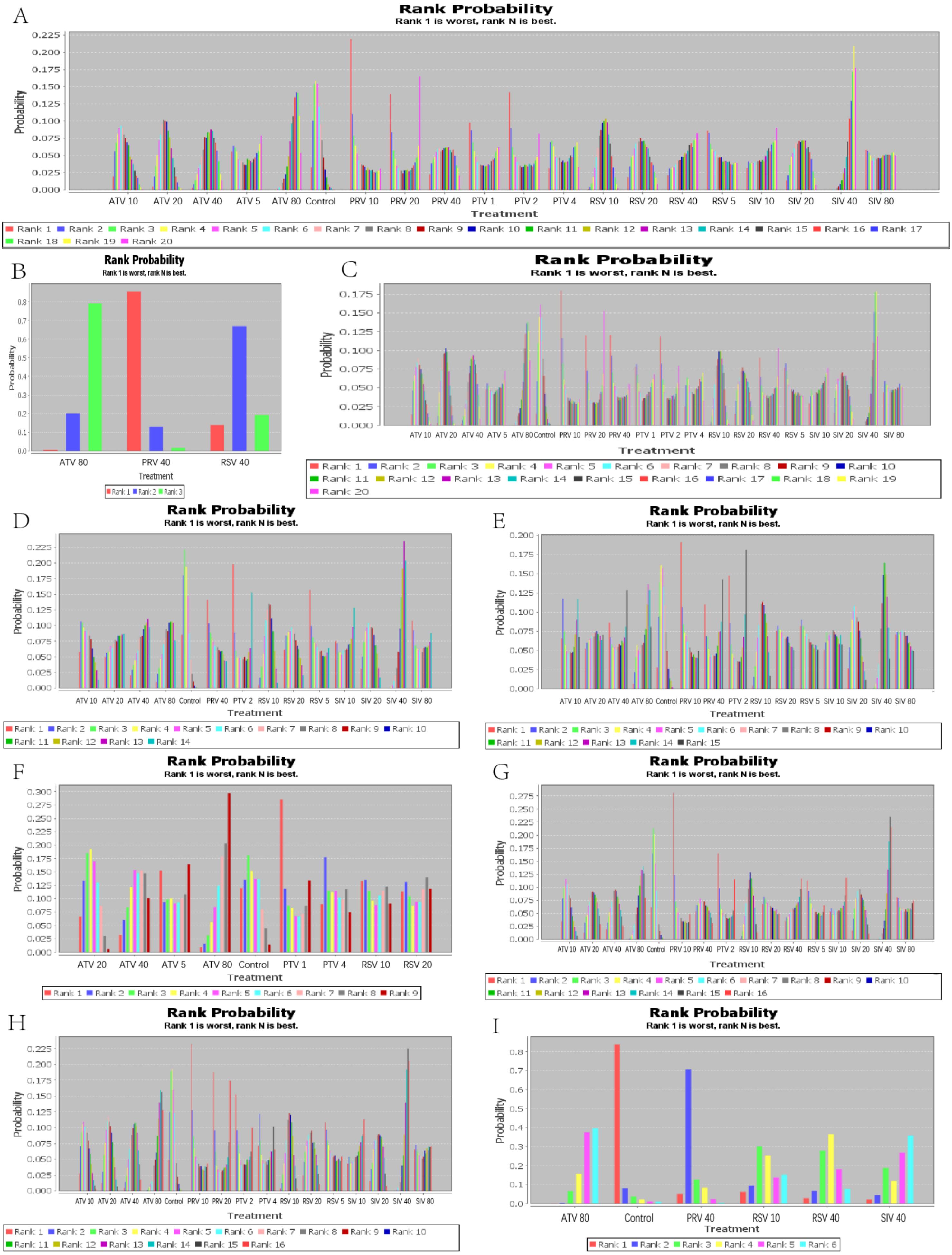

Supplementary Figure 1 | Results of ranks for various types and doses of statins. Notes: A: all included studies; B: subgroup analysis of CRP; C: subgroup analysis of hs-CRP; D: subgroup analysis of CRP/hs-CRP with clear measurement method; E: subgroup analysis of dyslipidemia; F: subgroup analysis of ACS; G: subgroup analysis of non-ACS; H: subgroup analysis of $<$12-month duration; I: subgroup analysis of $\geq$12-month duration.
